# Lycorine hydrochloride inhibits cell proliferation and induces apoptosis through promoting FBXW7-MCL1 axis in gastric cancer

**DOI:** 10.1186/s13046-020-01743-3

**Published:** 2020-10-30

**Authors:** Chongyang Li, Chaowei Deng, Guangzhao Pan, Xue Wang, Kui Zhang, Zhen Dong, Gaichao Zhao, Mengqin Tan, Xiaosong Hu, Shaomin Shi, Juan Du, Haoyan Ji, Xiaowen Wang, Liqun Yang, Hongjuan Cui

**Affiliations:** 1 State Key Laboratory of Silkworm Genome Biology, College of Biotechnology, Southwest University, #1, Tiansheng Rd., Beibei District, Chongqing, 400716 China; 2 Cancer center, Medical Research Institute, Southwest University, Chongqing, 400716 China; 3 Chongqing Engineering and Technology Research Centre for Silk Biomaterials and Regenerative Medicine, Chongqing, 400716 China; 4 Engineering Research Center for Cancer Biomedical and Translational Medicine, Southwest University, Chongqing, 400716 China; 5 Chongqing General Hospital, University of Chinese Academy of Sciences, Chongqing, 400014 China; 6 The Fifth Hospital of Shijiazhuang, Shijiazhuang, 050021 China; 7 The Third Hospital of Hebei Medical University, Shijiazhuang, 050051 China

**Keywords:** Gastric cancer, Lycorine hydrochloride, MCL1, FBXW7, Apoptosis, Cell cycle, Drug-resistance, PDX model

## Abstract

**Background:**

Lycorine hydrochloride (LH), an alkaloid extracted from the bulb of the *Lycoris radiata*, is considered to have anti-viral, anti-malarial, and anti-tumorous effects. At present, the underlying mechanisms of LH in gastric cancer remain unclear. MCL1, an anti-apoptotic protein of BCL2 family, is closely related to drug resistance of tumor. Therefore, MCL1 is considered as a potential target for cancer treatment.

**Methods:**

The effect of LH on gastric cancer was assessed in vitro (by MTT, BrdU, western blotting…) and in vivo (by immunohistochemistry).

**Results:**

In this study, we showed that LH has an anti-tumorous effect by down-regulating MCL1 in gastric cancer. Besides, we unveiled that LH reduced the protein stability of MCL1 by up-regulating ubiquitin E3 ligase FBXW7, arrested cell cycle at S phase and triggered apoptosis of gastric cancer cells. Meanwhile, we also demonstrated that LH could induce apoptosis of the BCL2-drug-resistant-cell-lines. Moreover, PDX (Patient-Derived tumor xenograft) model experiment proved that LH combined with HA14–1 (inhibitor of BCL2), had a more significant therapeutic effect on gastric cancer.

**Conclusions:**

The efficacy showed in our data suggests that lycorine hydrochloride is a promising anti-tumor compound for gastric cancer.

## Background

Gastric cancer, a malignant tumor originating from the epithelium of gastric mucosa, affects the health of nearly 1 million individuals every year [1]. The high mortality rate associated with gastric cancer (nearly 800,000 deaths per year) is mainly due to delayed diagnosis and limited treatment options [2, 3]. Although some progress has been made in the prevention, early diagnosis and effective treatment of gastric cancer, the prognosis of gastric cancer is still unsatisfactory [4–6]. For approximately 80% of gastric cancer patients, the diagnosis is lagged, and the relapse is frequent after surgery. Standard surgical resection is not ideal for advanced gastric cancer treatment. Therefore, the screening of new drugs is particularly urgent [7]. Besides, another crucial problem we have to face is that multi-drug-resistance has become one of the thorniest obstacles to the success of cancer chemotherapy [8, 9]. Accordingly, the development of effective inhibitors for drug-resistance-targets is also insistent.

Multiple shreds of evidence have shown that the chemo-resistance is intimately related to the intrinsic apoptosis regulators [10–13]. The BCL2 family proteins are the essential regulators of apoptosis and play vital roles in maintaining the physiological differentiation of cells and the dynamic balance of cell numbers. The members of the BCL2 family have conservative BCL2 homology (BH) domain sequences (BH1-BH4), which can be divided into two groups with completely opposite functions. The anti-apoptosis proteins, including BCL2, BCL-XL, BCL-W, BFL-1/A1 and MCL1, promote cell survival. Conversely, the anti-survival proteins, including BIM, BID, PUMA, NOXA, BAD, BMF, HRK and BIK, promote cell apoptosis [14, 15]. When the apoptosis signal is triggered, a subset of anti-survival proteins (like BIM, NOXA and PUMA) which have the BH3-only region, cause BAK and BAX homologous oligomerization and form pores in the mitochondrial membrane, leading to the release of Cytochrome C into the cytosol and further triggering apoptosis [16]. In this process, anti-apoptotic proteins (like BCL2, BCL-XL and MCL1) dynamically regulate apoptosis by binding or sequestering with the BH3-only domain proteins [17].

Considering the critical functions of the BCL2 family in cancer therapy, researchers have developed a large number of small molecule inhibitors over the past 10 years. ABT-737, the first BH3 mimetic inhibitor of BCL2, BCL-XL and BCL-W, exhibits favorable single-agent anti-tumorous activity in various tumor models [18]. Subsequently, ABT-263 (the upgraded products of ABT-737), BM 1197, S44563, BCL2 32, and AZD4320, as inhibitors of BCL2 and BCL-XL, were also reported to inhibit cancer progression successively [18, 19]. Further, the effects of mono-selective BCL2 inhibitors such as ABT-199 (also known as Venetoclax) and S55746 (also called BCL201 or Servier-1) were also reported in cancer research [20]. In previous clinical studies, ABT-263 showed single-drug efficacy in a variety of tumor types [21]. However, amplification of MCL1 is a potent factor of resistance to ABT-263 and ABT-737. Besides, some agents, synergistically promoting the degradation of MCL1, have been reported to induce apoptosis in many types of cancer cells [22]. At present, various kinds of BCL2 inhibitors have been developed and clinically tested, but MCL1 inhibitors are not available in clinical trials [23]. In addition, MCL1 is an essential cause of resistance to radiation and chemotherapy, including inhibitors targeting the other BCL2 family members [24]. For example, BCL2 selective inhibitor ABT-199 has shown high efficacy in the treatment of chronic lymphocytic leukemia (CLL), but it cannot induce apoptosis in certain tumor cell lines with MCL1 amplification [25]. Therefore, the development of effective MCL1 inhibitors has become an urgent necessity for clinical treatment.

Lycorine hydrochloride (LH), a derivative of lycorine, is an isoquinoline alkaloid extracted from *Lycoris*. According to previous reports, lycorine has a variety of pharmacological activities including anti-tumor, anti-virus, anti-inflammatory, anti-malaria, inhibition of acetylcholinesterase activity, etc. [26–28]. It has been reported that lycorine and its derivatives have significant inhibitory effects on leukemia, lymphoma, melanoma, esophageal cancer, breast cancer, ovarian cancer, prostate cancer, etc. [25]. So far, the existing evidence showed that LH has stronger therapeutic effect on tumor cells than normal cells [29]. However, LH has rarely been reported in gastric cancer. Therefore, it is necessary to study the impact of LH on gastric cancer and explore the underlying mechanisms.

## Materials and methods

### Reagents and antibodies

The MCL1 (16225–1-AP), HA (51064–2-AP), Alpha Tubulin (11224–1-AP) and FBXW7 (28424–1-AP) antibodies were purchased from Proteintech Group (Wuhan, China). The CDK1 (#77055), CDK2 (#78B2), Cleaved Caspase 3 (#14220), Cleaved Caspase 9 (#52873), Cleaved PARP (#5625), PARP (#9542), Bim (#2933), BAX (#5023) and BCL2 (#15071) antibodies were purchased from Cell Signaling Technology (CST, Boston, MA, USA). Cycloheximide (C7698), 5-Bromo-2-deoxyuridine (BrdU), 3-(4, 5-Dimethylthiahiazol-2-yl)-2, 5-diphenyltetrazolium bromide (MTT, M5655), dimethyl sulfoxide (DMSO, D5879) and Z-Leu-leu-leu-al (MG132, M7449) were purchased from Sigma-Aldrich (St. Louis, MO, USA). The anti-BrdU (ab8152) and Ki67 (ab15580), β-TrCP (ab71753) and HUWEI/mule (ab70161) antibodies were purchased from Abcam (Cam-bridge, MA, USA). The One Step TUNEL Apoptosis Assay Kit (C1089), RIPA lysis buffer, Phenylmethanesulfonyl fluoride (PMSF) and BCA protein assay kit were purchased from Beyotime (Shanghai, China). Lycorine hydrochloride was purchased from MUST BIO-TECHNOLOGY (Cheng Du, China). Alexa Fluor 488 goat anti-rabbit IgG (H + L) (35552) was purchased from Invritrogen (California, USA). 2-(4-Amidinophenyl)-6-indolecarbamidine dihydrochloride (DAPI), puromycin (A1113803) were purchased from Life Technologies (New York, USA). The transfection reagent Lipofectamine™ 2000 was obtained from Thermo Fisher Scientific (New York, USA). HRP goat anti-mouse and goat anti-rabbit antibodies were purchased from Beyotime (Shanghai, China). Annexin V-APC (2005128) and propidium iodide (2048964) were obtained from Invitrogen (California, USA). Super ECL prime (Cat: S6008-100 mL) were purchased from US Everbright®Inc. (Suzhou, China).

### Cell culture

MKN-45, SGC-7901 and 293-FT cell lines were purchased from American Type Culture Collection (ATCC, Manassas, VA, USA). All the cell lines were free of mycoplasma contamination as tested by vendors using MycoAlert kit from Lonza. MKN-45-R and SGC-7901-R cell lines were obtained from our laboratory. MKN-45, SGC-7901, MKN-45-R and SGC-7901-R cell lines were cultured in 1640 medium with 1% Penicillin-Streptomycin Solution and 10% fetal bovine serum (Biological Industries, BI, Beit HaEmek, Israel). 293-FT cells were cultured in DMEM medium with 2% glutamine (Biological Industries, Connecticut, USA), 1% non-essential amino acids (Biological Industries, Connecticut, USA), 1% sodium pyruvate (Biological Industries, Connecticut, USA), and 10% fetal bovine serum (BI). The above cell lines were cultured in standard conditions (5% CO_2_, 37 °C).

### Cell viability detection

MTT assay was performed to detect cell viability as previously described [30]. Simply put, SGC-7901 and MKN-45 cells in logarithmic phase were counted and seeded in 96 well plates (800 cells in 200 μL medium per well) and then attached overnight. RPMI-1640 complete medium with different concentrations LH (10, 20 and 40 μM) were used to treat SGC-7901 and MKN-45 cells, respectively. DMSO was used as control. MTT (5 mg/mL, 20 μL per well) was added into the culture medium at the designated time points and cultured at 37 °C incubator for 2 h. Formazan was further dissolved with DMSO (200 μL). The absorbance at 560 nm was monitored by the microplate reader (Thermo Fisher, Waltham, Ma, USA). All the experiments were carried out independently in triplicates. The data were analyzed by the Graphpad.

### BrdU staining

BrdU staining was used to monitor the cell proliferation as previously described method [20]. 2 × 10^4^ SGC-7901 or MKN-45 cells in logarithmic phase were inoculated into 24 well plates and cultured overnight in 37 °C incubator. LH (20 μM) was then used to treat gastric cancer cells. DMSO was applied as control. After 48 h, 10 μg/mL of BrdU was added into the medium for 2 h, and then 4% paraformaldehyde was used to fix cells for 15 min. After treatment with 2 M HCL and 0.3% Triton X-100, the cells were blocked with 10% goat serum (zsgb bio, Beijing, China). The cells were then successively incubated with BrdU primary antibody and then with secondary antibody. Before microscopic observation, the cells were stained with DAPI. BrdU positive cells were counted in three random areas.

### Western blotting assay

Cells were collected and then lysed in a RIPA lysis buffer with phenylmethanesulfonyl fluoride (PMSF) as previously described [31]. Protein concentration was detected with a BCA protein assay kit. The protein was separated on 8–12% SDS-PAGE Gels, and then transferred to a polyvinylidene fluoride (PVDF) membrane. After being blocked with 5% BSA at room temperature for 2 h, the PVDF membrane was incubated with a diluted primary antibody overnight at 4 °C. The next day, the PVDF membranes were incubated with horseradish peroxidase-conjugated secondary antibody (HRP-conjugated secondary antibodies) at room temperature for 2 h. Finally, the results were analyzed with the Super ECL prime (US Everbright®Inc., Suzhou, China) and western blotting detection system (Qin Xiang, Shanghai, China).

### Quantitative and reverse transcriptional PCR

After treatment with DMSO or LH at 37 °C for 48 h, cells were harvested and total RNA was isolated from cells using Trizol reagent according to the manufacturer’s instructions as previously described [32]. Total RNA was reverse-transcribed to cDNA using M-MLV reverse transcriptase (Promega, Wisconsin, USA). The qRT-PCR was performed in 20 μL reaction mixture, containing 2 μL cDNA template, 10 μL 2 × GoTaq® qPCR Master Mix (Promega, USA), 0.5 μL forward primers (Huada Gene, China), 0.5 μL reverse primers (Huada Gene, China) and 7 μL nuclease-free water. The amplification program went as follows: 95 °C for 5 min, followed by 45 cycles of 95f for 15 s, 60 °C for 30 s and 72 °C for 90 s, then 72 °C extension for 10 min. The normalized expression control was based on the glyceraldehyde-3-phosphate dehydrogenase (GAPDH) value. Then, the relative mRNA expression levels were quantified by using the 2^-∆∆Ct^ method. All primers were showed in Table 1. All the qRT-PCR results were repeated three times.

**Table 1:**
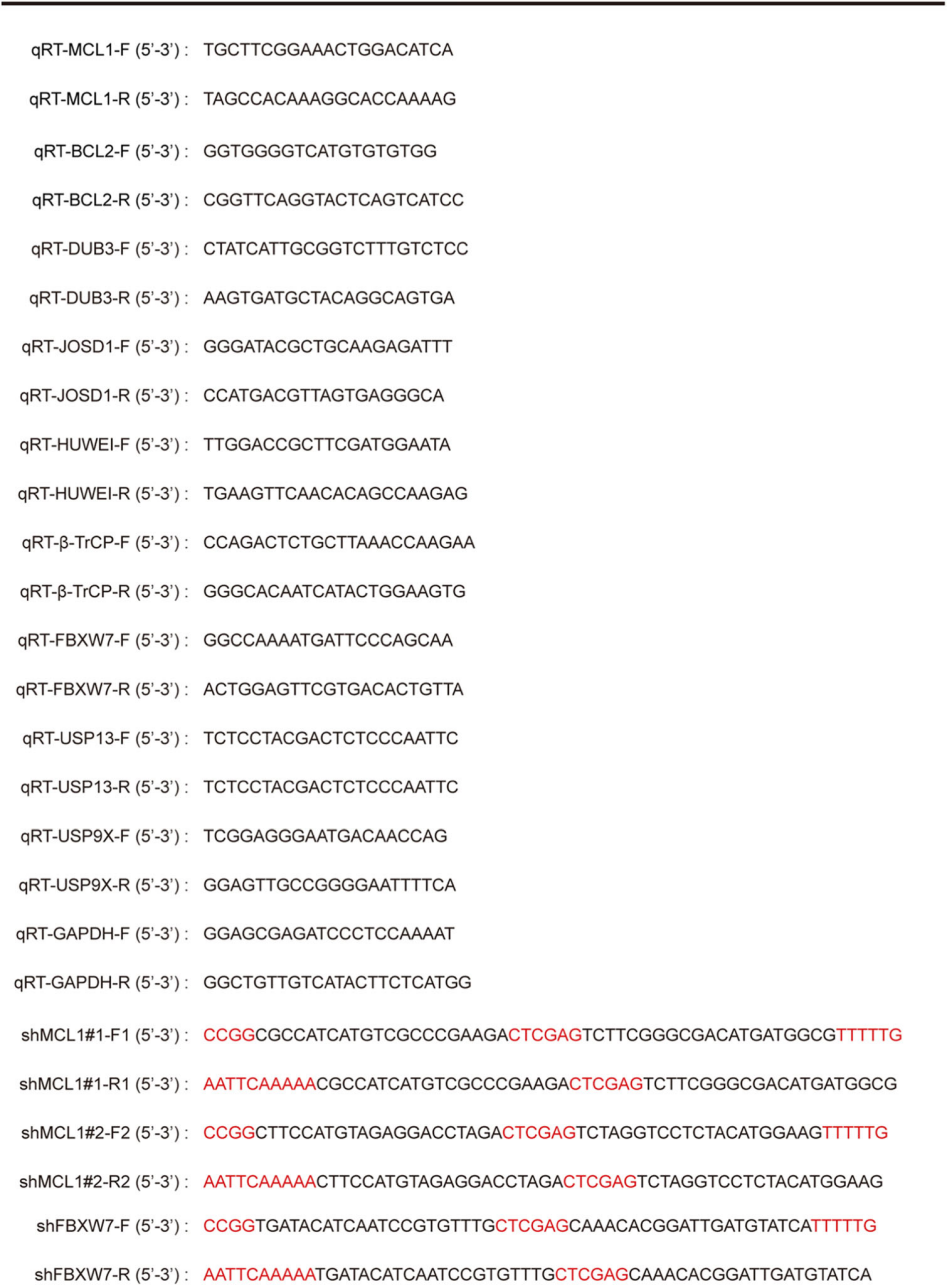
Primer sequences

### Flow cytometry assay

- Cell cycle analysis: MKN-45 and SGC-7901 cells were harvested after treatment with LH (20 μM) for 48 h, and fixed in 70% ethanol at 4 °C for 24 h. After being washed with PBS, the cells were added 2 μL RNaseA (4 mg/mL) and 5 μL propidium iodide for 35 min at 37 °C. Subsequently, the BD Accuri C6 flow cytometer and FlowJo software were used to analyze the change of DNA.
- Cell apoptosis analysis: MKN-45 and SGC-7901 cells were harvested after treatment with LH for 48 h. After being washed with 1 × Bingding buffer, the cells were incubated with Annexin V-APC (5 μL) and propidium iodide (5 μL) at room temperature for 20 min. Subsequently, the BD Accuri C6 flow cytometer and FlowJo software were used to analyze the change of apoptosis following the manufacturer’s instructions.

### TUNEL experimental analysis

Twenty thousand cells were seeded on the 24-well cell culture plate. After 24 h, LH (20 μM) and DMSO were added respectively, and incubated for 48 h. The PFA (4%) was used to fix the cells for 30 min. After being added PBS containing 0.5% Triton X-100 and incubated at room temperature for 5 min, the cells were added the prepared TUNEL test solution (50 μL/well) at 37 °C incubated for 60 min. Finally, the result was observed by fluorescence microscope after sealing with anti-fluorescence quenching solution. The excitation wavelength of Cy3 is 550 nm, and the emission wavelength is 570 nm.

### Transfection and infection

The plasmid shMCL1 (#1, #2), shFBXW7 and the negative control (SHC002) were purchased from Sigma-Aldrich (St. Louis, MO, USA). The MCL1-overexpression plasmid (PCDH-CMV-MCS-EF1-copGFP-MCL1) was purchased from YouBio (Changsha, China) [19]. Transfection and infection were carried out as manufacturer’s instructions. Firstly, the liposomes and the packaging plasmids (PLP1, PLP2, VSVG, target plasmids: shMCL1, shFBXW7 or OE-MCL1) were transferred into 293-FT cells. The virus was harvested 48 h later. Gastric cancer cell lines MKN-45, SGC-7901 were infected with the harvested virus.

### Subcutaneous tumor xenografts (CDX model)

Experiments in vivo were carried out with the approval of the Committee for Animal Protection and Utilization of Southwest University. According to the Guidelines for Animal Health and Use (Ministry of Science and Technology, China, 2006), all experiments were conducted orderly. Purchased from Huafukang Biotechnology Co., Ltd. (Beijing, China), six five-week-old female nude mice were raised and observed in SPF room for a week to adapt to the new environment. On June 20, 2019, SGC-7901 cells (1 × 10^6^ cells per mouse) suspended in 0.1 mL serum-free RPMI-1640 were subcutaneously inoculated on the left and right upper back of mice. In order to alleviate the pain of mice, we used the isoflurane for nasal anaesthesia before subcutaneous injections. Isoflurane can make the mice enter anesthesia state faster and recover quickly. After the anesthesia stops, the mice commonly wake up within 2 min, and the control of anesthesia depth was very easy. If the mice were found to be out of condition during the operation, the anaesthesia machine would be shut off immediately, and the mice would be rescued quickly. Hence, the safety of mice was guaranteed. Isoflurane can be completely discharged from the alveoli through respiration without affecting metabolism in vivo, and has no effect on the experimental results. In addition, isoflurane is widely used in animal experiments in the world. The mouse anaesthesia system was purchased from Reyward Life Technology Co., Ltd. (Shenzhen, China). All experiments were performed on a sterile workbench of an SPF room [33]. Before and after subcutaneous injection, 75% medical alcohol was used to disinfect the epidermis of mice. After 1 week injection, they were randomly divided into two groups, which were respectively treated with DMSO and LH (30 mg/kg) once a day for 16 days. During this period, tumor volume [tumor volume = (length×width^2^)/2] was measured every 2 days under strict and standardized feeding conditions. Before tumor collection, nasal anesthesia (isoflurane) was used in mice to relieve pain. Then, the mice were killed by cervical dislocation. The tumor was removed, and the weight was recorded. The bodies were frozen at − 20 °C before transferring to Laibite Biotech Inc. (Chongqing, China) for incineration. Finally, the tumor was photographed and recorded, which will be used for subsequent immunohistochemistry experiments.

### PDX experiment

Purchased from Huafukang Biotechnology Co., Ltd. (Beijing, China), 20 five-week-old female nude mice were raised and observed in SPF room for a week to adapt to the new environment. On August 5, 2020, with the consent of the patient’s family, the GAM-AD tumor mass from The Ninth People’s Hospital of Chongqing was cut into even 2 × 2 × 2 mm pieces and suspended in 0.1 mL serum-free RPMI-1640, and planted into subcutaneous tissue of mice in equal volume. The whole process was consistent with the previous subcutaneous tumor xenografts experiment. After 2 weeks, they were randomly divided into four groups, respectively treated with DMSO, LH (30 mg/kg) and LH (30 mg/kg) + HA14–1 (2.5 mg/kg) once a day for 13 days. In addition, the weight of the mice was recorded every 4 days. The process of tumor collection was also consistent with the aforementioned subcutaneous tumor xenografts experiment. The tumor weight was recorded, and the tumor was photographed and recorded.

### Jin’s formula

We evaluated the drug combined effects on anti-tumor through Jin’s formula [34]. The Jin’s formula was as follows:

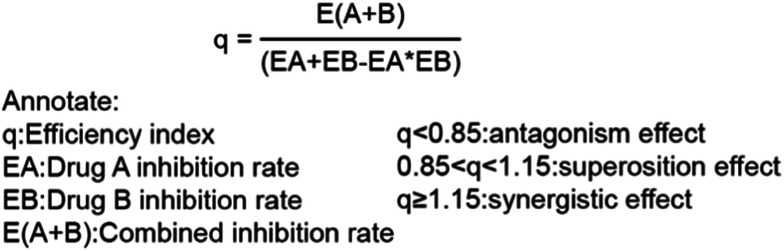



### Immunoprecipitation (co-IP)

Protein A/G Magnetic Beads (HY-K0202) were purchased from MCE (Monmouth Junction, NJ, USA). Cells treated with LH or DMSO were lysed and collected. Then, it was operated according to the manufacturer’s instructions.

### Ubiquitination assay

Firstly, the HA-Ub plasmid was transiently transferred into 293-FT. Then 293-FT cells were harvested before incubating with proteasome inhibitor MG132 (50 μg/mL) from Selleck (Houston, USA) for 6 h. The harvested cells were lysed by the IP lysis solution. Subsequently, it was incubated with anti-MCL1 (1%) or IgG at 4 °C for overnight. The second day, the Protein A/G Magnetic Beads were added following with the instruction. Then, the proteins adsorbed from magnetic beads were analyzed by western blotting. HA tag antibody (51064–2-AP) from Proteintech Group (Wuhan, China) was used to check the interaction between MCL1 and Ub.

### Immunohistochemistry assay

After the tumor tissues were paraffin sectioned, they were incubated with MCL1 (1:100) FBXW7 (1:100) or Ki67 (1:100) antibodies at 4 °C for overnight, then incubated with HRP-conjugated secondary antibodies for 20 min at room temperature. Subsequently, they were stained by DAB, and tissues were counterstained with hematoxylin. Finally, photographs were taken by the inverted microscope.

### The screen of BCL2-drug-resistant-cell-lines

The MKN-45 and SGC-7901 cell lines were cultured in 1640 complete medium with HA14–1(9 μM) for a week. Then the dead cells were washed with PBS. The remaining living cells were diluted in 96-well plate by gradient dilution method and maintained a high concentration of HA14–1. Continue to cultivate for 3 weeks. Finally, the surviving monoclonal cells were selected, and amplification cultured. Finally, the HA14–1-resistant cell lines (MKN-45-R and SGC-901-R) were obtained.

### Autophagy flux detection

The mRFP-GFP-LC3-adenovirus (HB-AP210 0001) was purchased from HanBio (Shanghai, China). Subsequently, mRFP-GFP-LC3B-adenovirus was added into the medium of MKN-45 and SGC-7901, cultured for 24 h. Then, the old medium was removed and fresh 1640 medium (with 10% fetal bovine serum, 1% Penicillin, Streptomycin, and 20 μM LH) was added. After 48 h, confocal microscopy was used to record the experimental results.

### Statistical analysis

Statistical analyses were obtained by the Graphpad prism software. All observations were confirmed by at least three independent experiments. Data were showed as MEAN ± SD and analyzed by unpaired 2-tailed t-test. *P*-values of < 0.05 (*), < 0.01 (**), and < 0.001 (***) were considered statistically significant.

## Results

### Lycorine hydrochloride inhibits gastric cancer cells growth and tumorigenesis

**Fig. 1:**
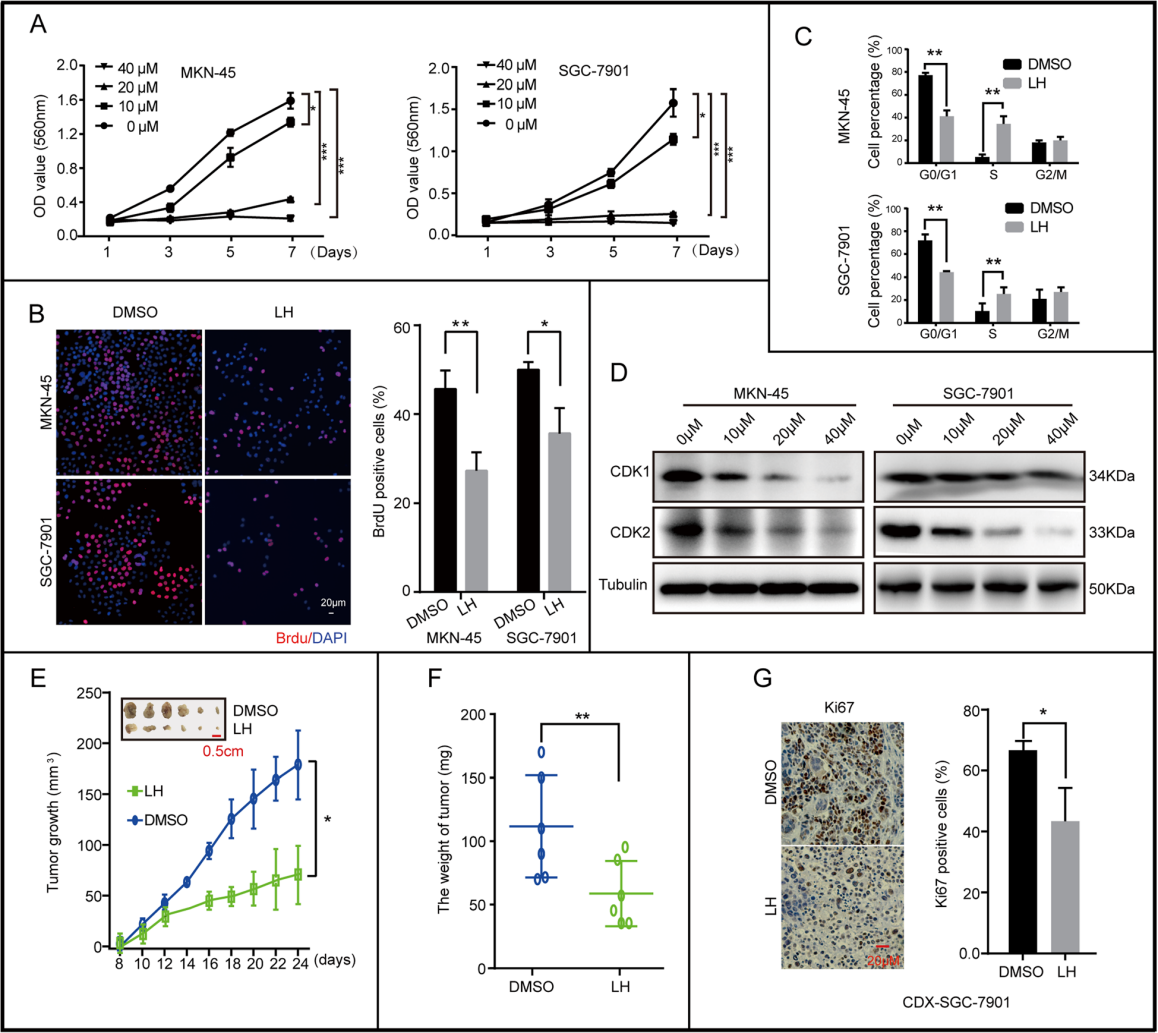
Lycorine hydrochloride inhibits gastric cancer cells growth and tumorigenesis. **a** Viability of MKN-45 and SGC-7901 cells after treatment with 10, 20, and 40 μM LH. DMSO was used as control. **b** BrdU-positive MKN-45 and SGC-7901 cells after treatment with 20 μM LH for 48 h. DMSO was used as control. The histogram demonstrated the results of the quantification of the number of BrdU-positive cells in MKN-45 and SGC-7901 cells. **c** Cell cycle of MKN-45 and SGC-7901 cells treated with 20 μM LH for 48 h were analyzed by flow cytometry. DMSO was used as control. Percentage indicated MKN-45 and SGC-7901 cells at different phase. **d** The expression of CDK1 and CDK2 in gastric cancer cells treated with different concentration of LH (0, 10, 20, 40 μM) for 48 h. Tubulin was used as internal reference. **e**, **f** Tumor volume and weight of indicated mice. DMSO and empty vector were used as control. Scale bar =0.5 cm. **g** IHC of Ki67 in indicated tumors. Scale bar = 20 μm. Gray value of IHC positive signal in panel was quantified. All data were analyzed by unpaired Student’s t-tests and were showed as the means ± SD. *p < 0.05, **p < 0.01, ***p < 0.001

To investigate the effect of LH on gastric cancer cells, we treated gastric cancer cell lines, MKN-45 and SGC-7901, with different concentrations of LH (10, 20 and 40 μM) for 48 h. DMSO was used as control. The structural formula of LH was showed in Fig. S1B. MKN-45 and SGC-7901 cells exposed to LH showed that the number of cell fragments and apoptotic bodies increased in the culture medium, and the cells decreased in size and shrunk in a dose-dependent manner (Fig. S1A). Cell viability was analyzed by MTT assay and BrdU staining. MTT assay showed that LH significantly inhibited cells growth (Fig. 1a), and its semi-lethal concentration (IC50) was approximately 20 μM. In consideration of the potential toxicities of LH, we chose 20 μM LH as an indicated concentration for further investigations. BrdU staining showed that DNA synthesis decreased after treatment with 20 μM LH for 48 h (Fig. 1b). We further examined the cell cycle to assess whether LH inhibited cell proliferation by causing cell cycle arrest. Flow cytometry analysis showed that LH could arrest cell cycle progression at S phase (Fig. 1c). Furthermore, the western blotting showed that LH could significantly inhibit the expression of CDK1 and CDK2 in a dose and time dependent manner (Fig. 1d, S1C). To further investigate the effects of LH in vivo, SGC-7901 cells were injected subcutaneously into 5-week-old female nude mice. The results demonstrated that mice injected with LH had smaller tumor volume and less tumor weight comparing with the mice injected with DMSO (Fig. 1e, f). Immunohistochemical (IHC) staining with Ki67 further supported the results that LH inhibits tumourigenecity in gastric cancer cells (Fig. 1g). In conclusion, LH could dramatically inhibit the growth and tumorigenesis of gastric cancer cells.

### Lycorine hydrochloride induces gastric cancer cells apoptosis

**Fig. 2:**
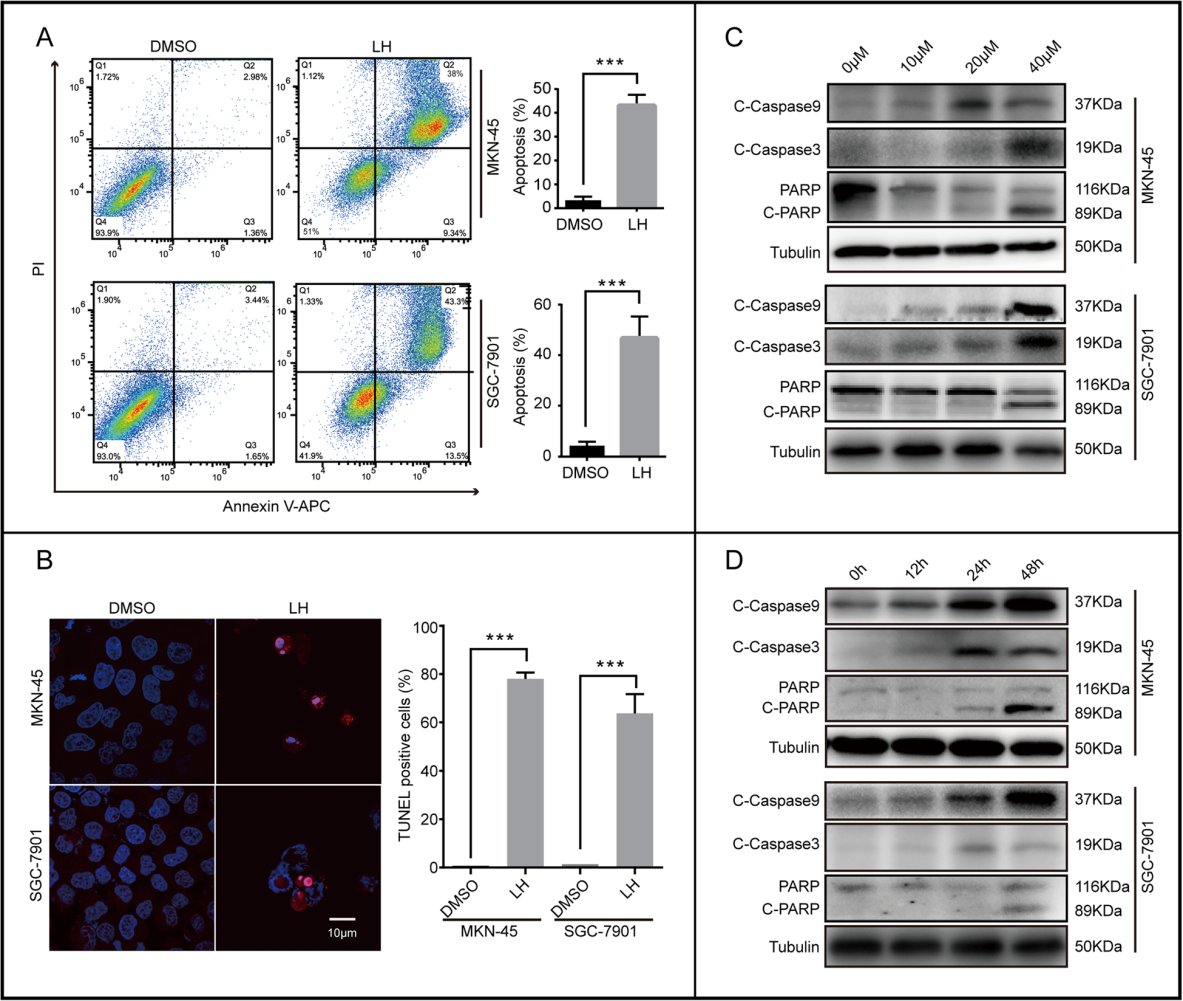
Lycorine hydrochloride induces apoptosis in gastric cancer cells. **a**, **b** Apoptosis of MKN-45 and SGC-7901 cells treated with 20 μM LH for 48 h were examined by flow cytometry and TUNEL staining. DMSO was used as control. **c**, **d** The expression of apoptotic protein, including C-Caspase 9, C-Caspase 3, PARP and cleaved PARP in gastric cancer cells treated with LH at different concentrations and time gradients. DMSO was used as control. Tubulin was used as internal reference. All data were analyzed by unpaired Student’s t-tests and were showed as the means ± SD. *p < 0.05, **p < 0.01, ***p < 0.001

In addition to inhibiting the proliferation of gastric cancer cells, could LH affect apoptosis or autophagy? The mRFP-GFP-LC3-adenovirus system confirmed that there was no obvious autophagy flux in gastric cancer cells after treatment with LH (Fig. S1D). By flow cytometry apoptotic analysis, we found that LH could significantly induce apoptosis of gastric cancer cells (Fig. 2a). Similarly, TUNEL staining showed that LH did induce obvious apoptosis (Fig. 2b). To further confirm these results, we tested the apoptosis-related markers, including Cleaved Caspase 3 (C-Caspase 3), Cleaved Caspase 9 (C-Caspase 9) and cleaved poly ADP-ribose polymerase (C-PARP). The western blotting results proved that C-Caspase 9, C-Caspase 3 and C-PARP increased in a concentration and time dependent manner (Fig. 2c, d).

### Overexpression of MCL1 rescues cells proliferation and decreases apoptosis induced by lycorine hydrochloride

**Fig. 3:**
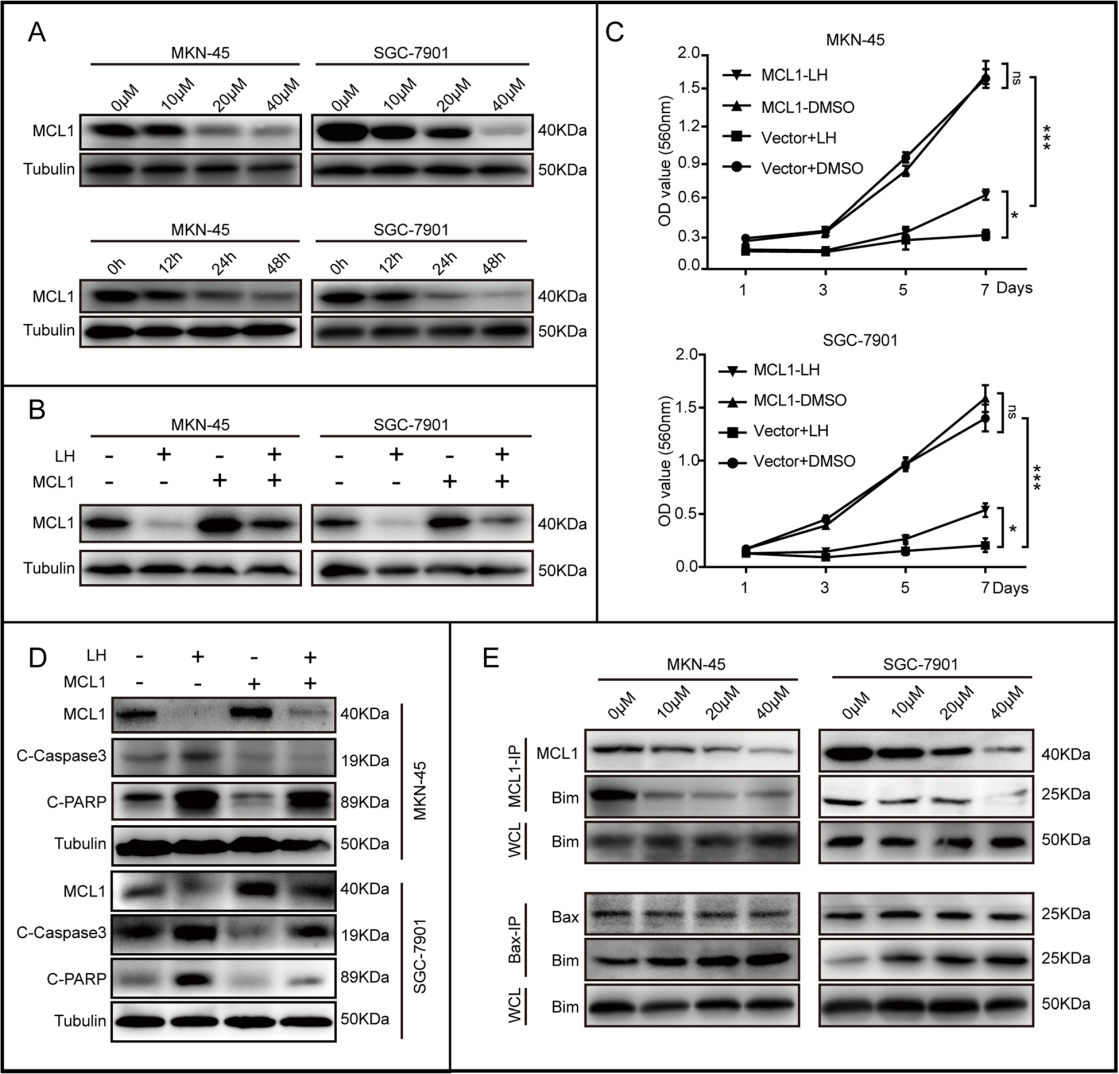
Overexpression MCL1 restores cell proliferation and decreases apoptosis induced by lycorine hydrochloride. **a** The expression of MCL1 in MKN-45 and SGC-7901 cells treated with different concentration LH (10, 20 and 40 μM) for 48 h and treated with 20 μM LH for different time (0, 12, 24 and 48 h). DMSO was used as control. Tubulin was used as internal reference. **b** The expression of MCL1 in 20 μM LH-treated cells overexpressing MCL1 or empty vector. **c** Growth curve of MKN-45 and SGC-7901 cells overexpressing MCL1 after treatment with 20 μM LH. DMSO and empty vector were used as control. **d** The expression of MCL1, cleaved-PARP and C-Caspase3 were checked in MKN-45 and SGC-7901 cells overexpressing with MCL1 after treatment with 20 μM LH for 48 h. DMSO and empty vector were used as control. Tubulin was used as internal reference. **e** The interaction of MCL1 and BIM; BIM and BAX were detected after treating with different concentration LH (10, 20 and 40 μM) for 48 h by immunoprecipitation. DMSO was used as control. All data were analyzed by unpaired Student’s t-tests and were showed as the means ± SD. *p < 0.05, **p < 0.01, ***p < 0.001

According to the online database (https://www.cbligand.org/HTDocking/searchstruct.php), we observed that there might be docking sites between LH and MCL1 (Fig. S3A). As reported, MCL1 is highly expressed in gastric cancer, and the prognosis of patients with high expression of MCL1 is worse [35]. After treatment with different concentration of LH (10, 20 and 40 μM, DMSO was used as control) for 48 h, we found that MCL1 was significantly decreased in a dose dependent manner in both MKN-45 and SGC-7901 cells (Fig. 3a). Moreover, MKN-45 and SGC-7901 cells were treated with 20 μM LH for 0, 12, 24 and 48 h. The results indicated that MCL1 was reduced in a time dependent manner as well (Fig. 3a). However, BCL2 and BAK did not markedly change with the addition of LH (Fig. S3B). By using lentivirus transfection system, we obtained stable MKN-45 and SGC-7901 cell lines with exogenous overexpression MCL1. Western blotting results demonstrated that MCL1 was up-regulated after infection with lentivirus. DMSO and empty vector were used as control (Fig. 3b). The MTT experiment also proved that the growth rate of overexpression MCL1 cells after LH treatment was significantly higher than the group of empty vector treated with LH (Fig. 3c). BrdU staining experiment illustrated that overexpression of MCL1 rescued the DNA synthesis decrease induced by LH (Fig. S3E). To further explore the effect of MCL1 on cell cycle arrest induced by LH, we performed flow cytometry experiment, of which results showed that MCL1 could partly rescue the cell cycle arrest caused by LH (Fig. S3F). Subsequently, western blotting results further showed that under the LH treatment, the related cyclins (CDK1 and CDK2) were partly restored after overexpression of MCL1 (Fig. S3G).

Meanwhile, the flow cytometry for apoptotic analysis and the TUNEL staining results indicated that overexpression of MCL1 decreased percentage of apoptotic cells induced by LH (Fig. S3C, D). Besides, western blotting was performed, and C-PARP and C-Caspase 3 were partially restored in the group of MCL1 overexpression (Fig. 3d). To further explore the mechanism of apoptosis, we carried out immunoprecipitation experiment, of which results showed that the interaction of MCL1 with BIM was decreased, and the interaction of BIM with BAX was raised after treatment with different concentration gradients LH for 48 h (Fig. 3e). In conclusion, the above results showed that MCL1 could partly rescue cell proliferation and decrease apoptosis induced by LH.

### Lycorine hydrochloride decreases the protein stability of MCL1 through FBXW7

**Fig. 4:**
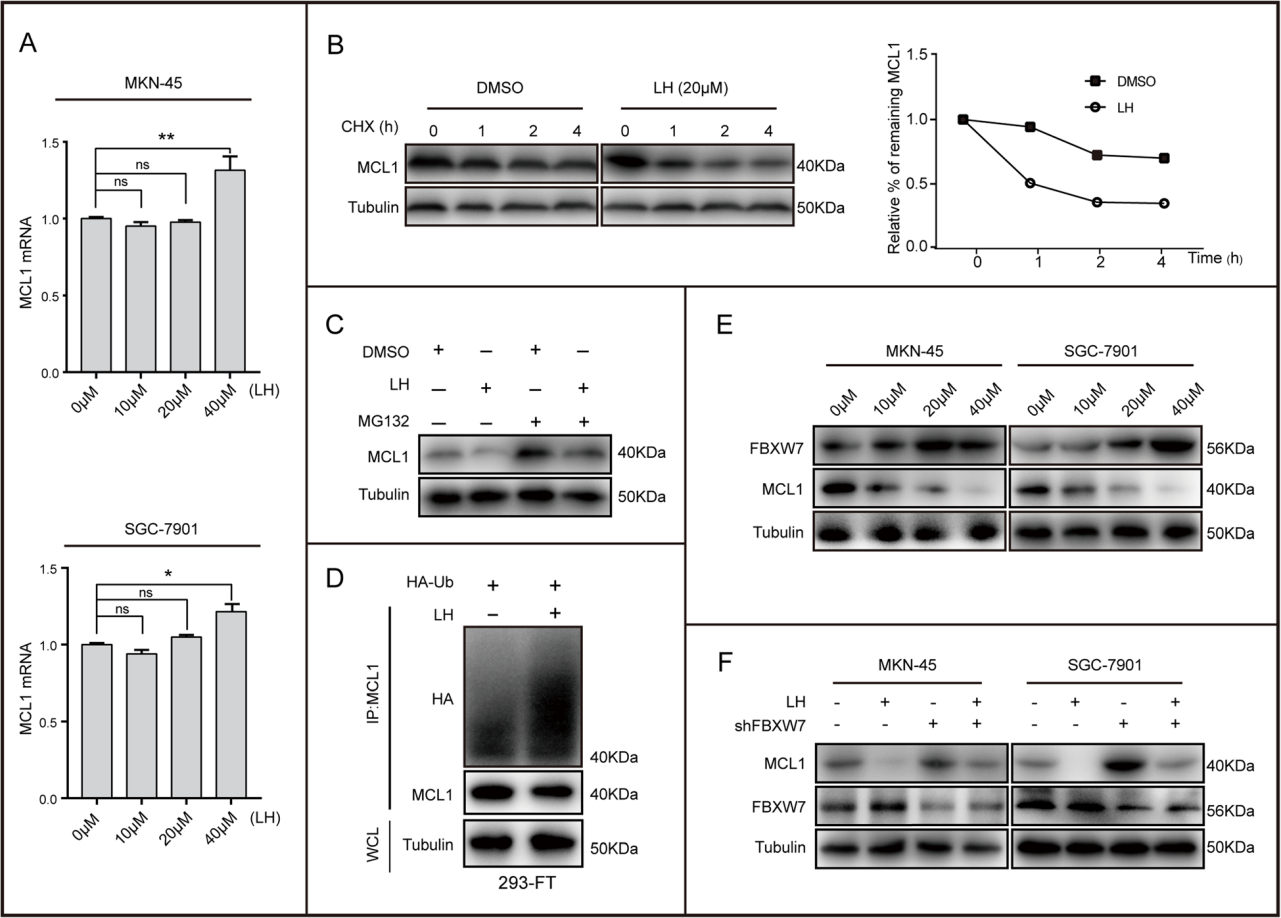
Lycorine hydrochloride affects the stability of MCL1 protein through FBXW7. **a** Quantitative PCR was performed to detect the mRNA level of MCL1 in gastric cancer cells after treatment with LH. **b** 293-FT cells were treated with LH (20 μM) or DMSO and were then treated with CHX (100 μg/mL) for the indicated times. Cell lysate was immunoblotted with the indicated antibodies. The density of MCL1 was measured, and the integrated optical density (IOD) was measured. The turnover of MCL1 was indicated graphically. **c** Cell lysate was prepared from 293-FT cells treated with DMSO or LH that had been treated with or without MG132 for 8 h. Equal amounts of cell lysate was immunoblotted with the indicated antibodies. **d** The ubiquitination of MCL1 in 293-FT cells was enhanced by treatment with LH. **e** Western blotting assays were performed to detect the expression of FBXW7 and MCL1 in MKN-45, SGC-7901 cells after treatment with LH. Tubulin was as internal reference. **f** Protein expression levels of MCL1 and FBXW7 were analyzed by western blotting in MKN-45, SGC-7901 cells. All data were analyzed by unpaired Student’s t-tests and were showed as the means ± SD. *p < 0.05, **p < 0.01, ***p < 0.001

To further explore the molecular mechanism of LH regulating MCL1, we performed qRT-PCR experiments. We found that the mRNA level of MCL1 did not decrease after treatment with LH, but increased slightly (Fig. 4a). Therefore, we speculated that LH might affect the protein stability of MCL1. Indeed, LH could decrease the turnover rate of MCL1 in the presence of the de novo protein synthesis inhibitor cycloheximide (CHX) (Fig. 4b). Meanwhile, the western blotting analysis revealed that the proteasome inhibitor MG132 could partly rescue the reduction of MCL1 after treatment with LH (Fig. 4c). Further, we found that LH could increase the ubiquitination levels of MCL1 (Fig. 4d). Therefore, we analyzed the related proteins which regulate the ubiquitination level of MCL1 by qRT-PCR. The primers used were listed in Table 1. The results showed that ubiquitin E3 ligase FBXW7 was up-regulated after treatment with LH (Fig. S4A). Furthermore, the western blotting showed that FBXW7 (not HUWEI or β-TrCP) was up-regulated after adding LH (Fig. 4e, S4B). After down-regulating FBXW7 in the gastric cancer cells, we found that the down-regulation of MCL1 expression induced by LH could be partially restored (Fig. 4f). Thereby, we concluded that LH could down-regulate the stability of MCL1 through FBXW7, promoting gastric cancer cells apoptosis, and inhibiting cells growth.

### Lycorine hydrochloride induces apoptosis of BCL2-drug-resistant gastric cancer cell lines

**Fig. 5:**
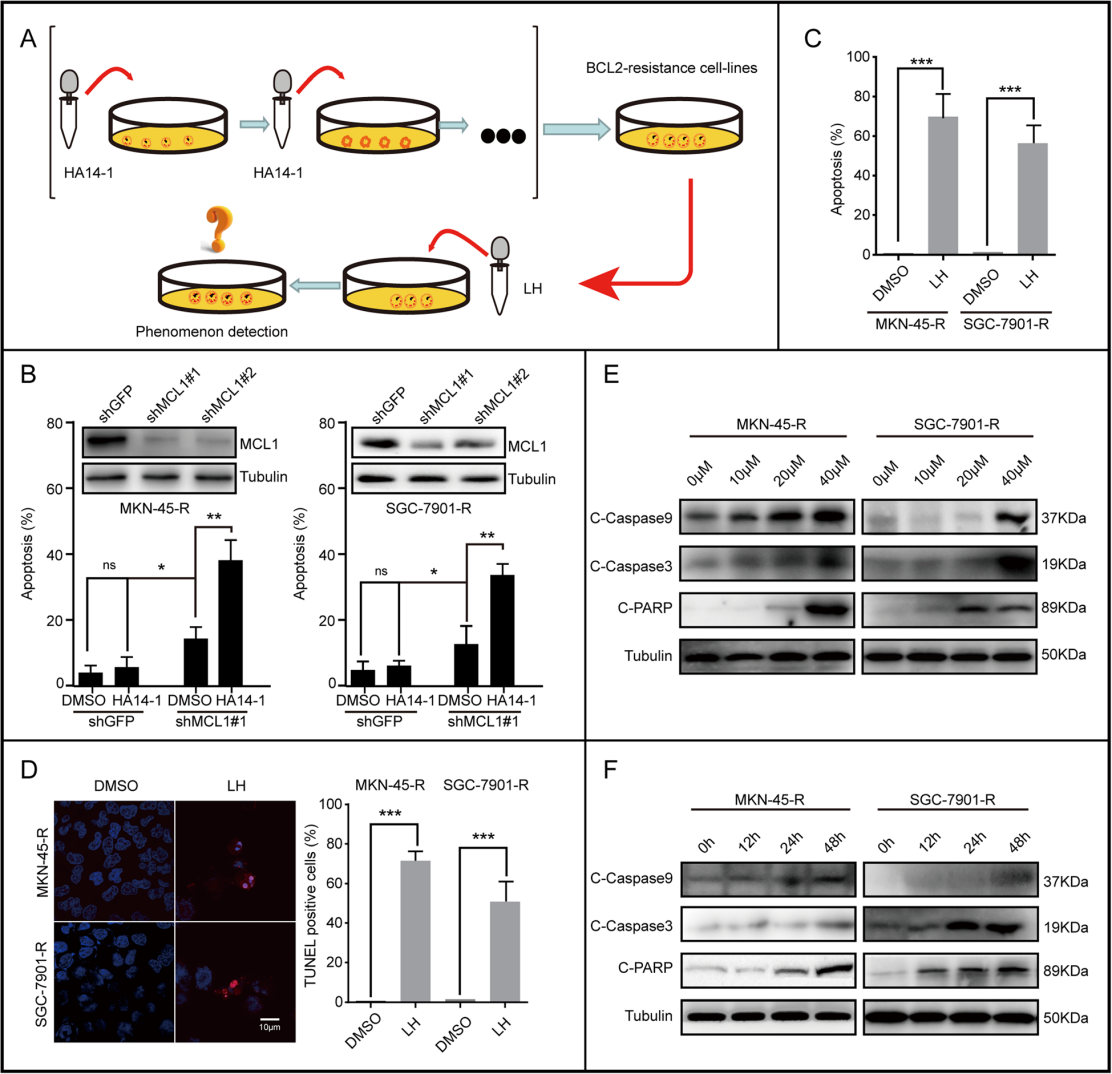
Lycorine hydrochloride induces apoptosis of BCL2-drug-resistant gastric cancer cell lines. **a** Screening of HA14–1 resistant cell lines (Detailed screening methods was described in the materials and methods). **b** Western blotting analysis of MCL1 expression in MKN-45-R and SGC-7901-R cell lines expressing shGFP, shMCL1#2, shMCL1#4. Trypan blue staining was used to analyze the apoptosis induced by DMSO or HA14–1 in MKN-45-R and SGC-7901-R cell lines expressing shGFP, shMCL1#1. **c**, **d** Apoptosis was analyzed in MKN-45-R, SGC-7901-R cells after treatment with 20 μM LH for 48 h by trypan blue and TUNEL staining. Apoptotic rate of MKN-45-R, SGC-7901-R cells in panel was quantified. **e**, **f** The expression of apoptotic protein, including C-Caspase 9, C-Caspase 3, PARP and C-PARP in BCL2-drug-resistant gastric cancer cells treated with LH at different concentrations and time gradients. DMSO was used as control. Tubulin was used as internal reference. All data were analyzed by unpaired Student’s t-tests and were showed as the means ± SD. *p < 0.05, **p < 0.01, ***p < 0.001

According to multiple lines of evidence indicated, MCL1 is commonly up-regulated in various cancers and is considered as a primary factor to resistance the treatment with the BCL2 inhibitor [36]. Besides, acquired resistance is an obstacle for most of drugs ever used in oncology. Our findings indicated that, with prolonged treatment of HA14–1, sensitive gastric cancer cell lines could spontaneously select to resistant it. So, can LH affect HA14–1-resistant cells? Based on our described screening method, we obtained drug-resistant-cell-lines (MKN-45-R, SGC-7901-R), which were resistant to HA14–1 (Fig. 5a). The IC50 of HA14–1 in BCL2-drug-resistant gastric cancer cell lines was higher than that in normal gastric cancer cell lines (Fig. S5A). Furthermore, the qRT-PCR and western blotting assays showed that the transcriptional and protein levels of MCL1 and BCL2 in BCL2-drug-resistant gastric cancer cell lines were higher than those in normal gastric cancer cell lines (Fig. S5B, C). MCL1 silencing promoted the apoptosis of drug-resistant-cell-lines, and the combination with HA14–1 (BCL2 specific inhibitor) significantly increased the apoptosis (Fig. 5b). The trypan blue staining and TUNEL staining showed that LH could also induce apoptosis in drug-resistant-cell-lines (Fig. 5c, d). Subsequently, western blotting showed that LH could also induce the up-regulation of apoptosis-related proteins, C-Caspase 9, C-Caspase 3 and C-PARP in BCL2-drug-resistant gastric cancer cell lines with a dose and time gradient effect (Fig. 5e, f). In brief, the above experiments showed that LH exactly has therapeutic effect on BCL2-resistant gastric cancer cell lines.

### The combination of lycorine hydrochloride and HA14–1 enhances the therapeutic effect on gastric cancer

**Fig. 6:**
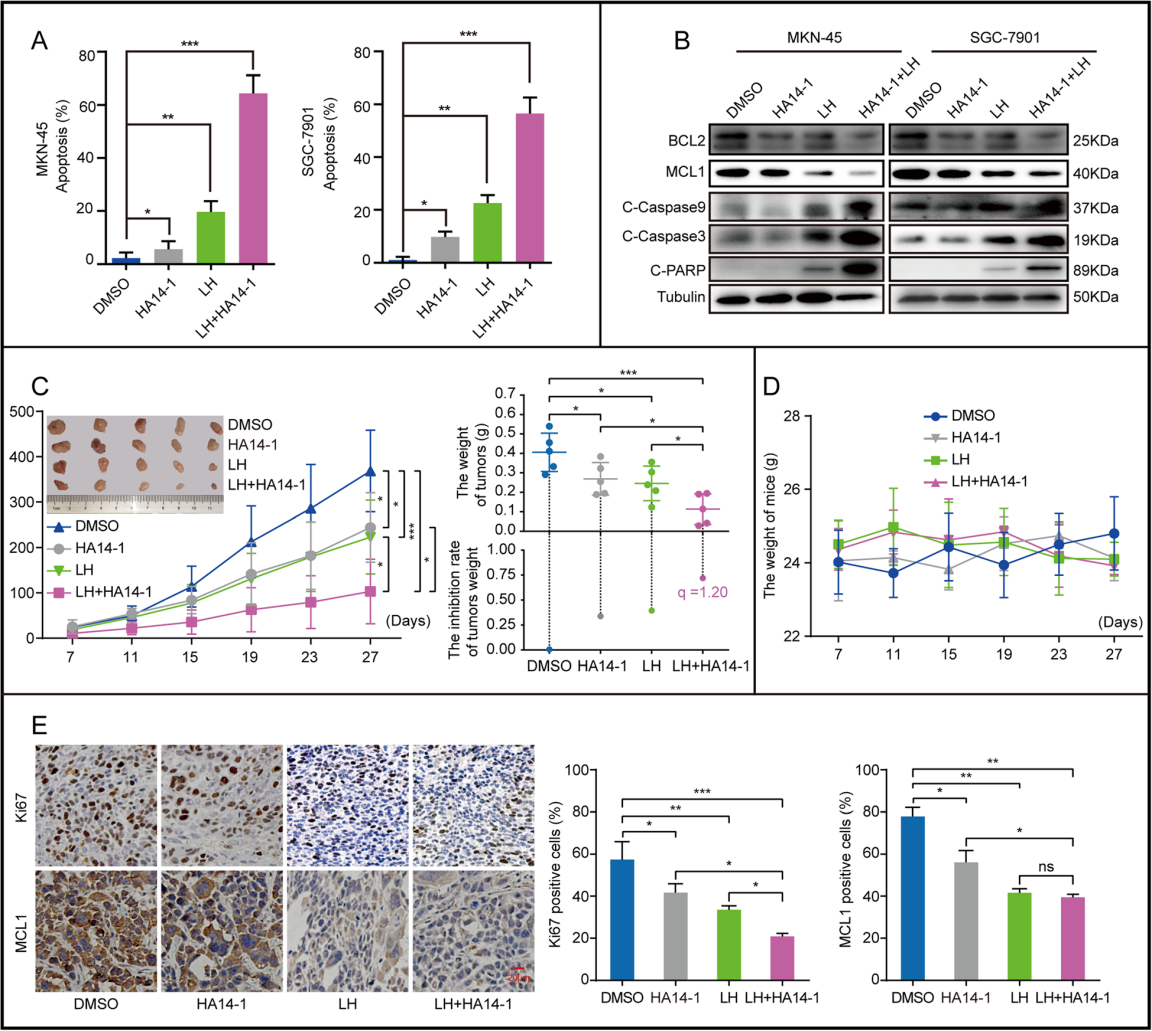
The combination of lycorine hydrochloride and HA14–1 enhances the therapeutic effect on gastric cancer. **a** Apoptosis of MKN-45 and SGC-7901 cells treated with LH (20 μM), HA14–1(9 μM) or LH (20 μM) + HA14–1(9 μM) for 48 h were examined by trypan blue staining. DMSO was used as control. Apoptotic rate of MKN-45 and SGC-7901 cells was quantified. **b** Western blotting was used to detect the expression of apoptotic protein, including BCL2, MCL1, C-Caspase 9, C-Caspase 3, PARP and C-PARP in MKN-45 and SGC-7901 cells after 48 h of treatment with LH (20 μM), HA14–1 (9 μM) or LH (20 μM) + HA14–1 (9 μM). DMSO was used as control. **c** Tumor volume and weight of indicated mice. DMSO was used as control. The efficiency index (q) analysis of LH (30 mg/kg) combined with HA14–1 (2.5 mg/kg) treatment in the weight of PDX tumors through Jin’s formula. **d** The weight of the mice treated with DMSO, HA14–1, LH or LH + HA14–1 was measured. **e** IHC of MCL1 and Ki67 in indicated tumors. Scale bar =20 μm. Gray value of IHC positive signal in panel was quantified. All data were analyzed by unpaired Student’s t-tests and are shown as the means ± SD. *p < 0.05, **p < 0.01, ***p < 0.001

According to our research, the trypan blue staining showed that HA14–1 and LH could induce apoptosis in gastric cancer cell lines, respectively (Fig. 6a). Besides, the apoptosis rate induced by the combination of the two drugs was significantly higher than that sum of the ratio of apoptosis induced by the two separated drugs (Fig. 6a). Similarly, western blotting results showed that the combination of LH and HA14–1 could significantly induce the expression of apoptosis-associated proteins such as C-Caspase 9, C-Caspase 3, and C-PARP (Fig. 6b). To further investigate the treatment effects of the combination of HA14–1 and LH in vivo, we carried out the PDX model experiment. Tumor masses (GAM-AD) from The Ninth People’s Hospital of Chongqing were transplanted subcutaneously into female nude mice. After 2 weeks, the mice were randomly divided into 4 groups, then separately injected with HA14–1 (2.5 mg/kg), LH (30 mg/kg), LH (30 mg/kg) + HA14–1 (2.5 mg/kg) and DMSO once a day. Measured every 4 days, the weight of the mice did not significantly differ between the control and drugs treatment groups (Fig. 6d), indicating that the drugs were hypotoxic in mice. However, the tumor weight was significantly decreased. In addition, compared with treatment with LH only, the treatment with LH + HA14–1 have a more obvious therapeutic effect (Fig. 6c). In order to better explain the effect of drug combination, we have conducted the efficiency index (q) analysis of LH (30 mg/kg) combined with HA14–1 (2.5 mg/kg) treatment in weight of PDX tumors through Jin’s formula. The results show that LH combined with HA14–1 exhibited obvious synergistic effects (q value≥1.15), rather than the superposition effect of the two drugs (Fig. 6c). Further, the IHC results showed that the Ki67 staining of LH treated mice was significantly reduced, and the expression of Ki67 was significantly decreased after treatment with LH + HA14–1 compared with treatment with LH only (Fig. 6e). Immunohistochemical (IHC) staining with MCL1 further supported the results that LH inhibited tumorigenicity in gastric cancer through down-regulating the expression of MCL1. In a word, the results indicated that LH combined with HA14–1 exhibited a more significant inhibitory effect than LH alone in vivo.

## Discussion

As one of the most common and deadly cancers in the world, gastric cancer is hard to cure, which is the third leading cause of cancer-related death in men and the fifth leading cause of cancer-related death in women [37]. We focused on the monomer of traditional Chinese medicine. As a tested drug, LH has not only low toxic side effects, but also the advantage of clear molecular formula. Through our study, we found that LH could inhibit the gastric cancer growth and induce apoptosis of gastric cancer cells.

Further, according to the database prediction (Fig. S3A), LH may have the possibility of interactions with MCL1. So we focused on MCL1, which is amplified in many types of tumor, such as lung, breast, prostate, pancreatic, ovarian and cervical cancers, melanoma and leukemia, etc. [38]. Besides, MCL1 has recently been regarded as a promising target for cancer treatment [38, 39]. In view of the previous reports, we knew that MCL1 is highly expressed in gastric cancer, and the prognosis of patients with high expression of MCL1 is worse [35]. In this study, LH was proved to cause cell cycle arrest at S phase and induced apoptosis in gastric cancer by inhibiting MCL1, which is consistent with the previous reports that MCL1 is involved in cell cycle by improving the stability of CDK2 protein [20]. Furthermore, we further confirmed that LH did not down-regulate the mRNA level of the MCL1. MCL1 protein is extremely unstable, with a very short half-life [40]. MCL1 degradation is regulated by its phosphorylation at several sites, leading to subsequent ubiquitination by E3 ligases such as F-box and WD repeat domain-containing 7 (FBXW7), HUWEI/Mule, and β-TrCP [41–45]. In addition, the stability of MCL1 protein is also regulated by deubiquitinase such as: JOSD1, DUB3, USP13, USP9X [46–49]. Therefore, we speculated whether LH affected the stability of MCL1 by directly binding, indirectly regulating, or the above two ways. Subsequently, qRT-PCR and western blotting experiments showed that FBXW7 could respond to LH and has a negative correlation with MCL1 expression. After FBXW7 was down-regulated, it could obviously save the down-regulation of MCL1 caused by LH. Hence, we suggested that LH may mainly regulate the proliferation and apoptosis of gastric cancer by regulating FBXW7-MCL1 axis. Moreover, the direct combination between LH and MCL1 needs further verification.

According to the previous report, overexpression of MCL1 is the cause of drug resistance of several chemotherapeutic agents. For example, overexpression of MCL1 induces resistance to many widely used anti-cancer therapies drugs such as BCL2 inhibitors, such as paclitaxel, vincristine, and gemcitabine [45, 50]. In this study, we screened BCL2-drug-resistant-cell-lines (MKN-45-R and SGC-7901-R). The qRT-PCR assay showed that the transcriptional level of MCL1 in BCL2-drug-resistant gastric cancer cell lines was higher than that in normal gastric cancer cell lines (Fig. S5). Besides, the apoptosis of the drug-resistant gastric cancer cell lines could also be induced by down-regulating MCL1 or adding LH. These results confirmed that LH not only induces apoptosis of gastric cancer but also may be a potential therapeutic drug for patients with BCL2-drugs- resistance. There is no doubt that BCL2 and MCL1, two anti-apoptotic members of the BCL2 family, have similar structures. The down-regulation of the BCL2 or MCL1 could counteract the apoptosis by up-regulation of the other one. In our study, we found that the combination of BCL2 and MCL1 inhibitors could induce gastric cancer cells more significantly apoptosis. The combination of LH and HA14–1 induced remarkable tumor growth inhibition in our PDX model.

## Conclusions

Taken together, in this study, lycorine hydrochloride (LH), an extract of *Lycoris radiate*, was proved to reduce the accumulation of MCL1 through FBXW7-MCL1 axis and induce apoptosis of gastric cancer cells. Besides, as a potential inhibitor of MCL1, LH could kill the BCL2-drug-resistant-cell-lines. Meanwhile, PDX model experiment showed that LH combined with HA14–1 greatly inhibited the growth of gastric cancer in vivo. In sum, the above results showed that LH is worthy being further researched as a clinical drug for gastric cancer treatment, and these findings provided a theoretical basis for the development of clinical drugs targeting MCL1 in gastric cancer.

## Supplementary Information

**Additional file 1: Figure S1.** Lycorine hydrochloride inhibits cell proliferation in gastric cancer cells. (A) Morphological changes of MKN-45 and SGC-7901 cells after treatment with 10, 20, and 40 μM LH were observed. DMSO was used as control. (B) The molecular structure formula of LH. (C) The expression of CDK1 and CDK2 in gastric cancer cells after treatment with 20 μM LH for different time (0 h, 12 h, 24 h and 48 h). Tubulin was used as internal reference. (D) Autophagy flux was detected by using the mRFP-GFP-LC3-adenovirus system. CQ (Chloroquine) was used as a positive control.

**Additional file 2: Figure S3.** Overexpression MCL1 decreases apoptosis and restores cell proliferation induced by lycorine hydrochloride. (A) The prediction docking score of LH to its target molecules (BCL2 family) were analyzed. (B) Western blotting to verify the predicted results (LH and its target molecules). (C, D) Apoptosis was analyzed in MKN-45 and SGC-7901 cells overexpressing MCL1 after treatment with 20 μM LH for 48 h by flow cytometry and TUNEL. LH + empty vector were used as control. Apoptotic rate of MKN-45 and SGC-7901 cells in histogram was quantified. (E) BrdU-positive cells in MCL1-overexpression MKN-45 and SGC-7901 cells after treatment with 20 μM LH. DMSO and empty vector were used as control. The histograms of BrdU positive MKN-45 and SGC-7901 cells were analyzed quantitatively. (F) Cell cycle in MKN-45 and SGC-7901 cells overexpressing MCL1 after treatment with 20 μM LH for 24 h. DMSO and empty vector were used as control. Percentage of MKN-45 and SGC-7901 cells from panel at different phase was analyzed quantitatively. (G) The expression of CDK1 and CDK2 together with MCL1 were checked in MCL1-overexpressed MKN-45 and SGC-7901 cells with 20 μM LH treatment for 48 h. DMSO and empty vector were used as control. Tubulin was used as internal reference. All data were analyzed by unpaired Student’s t-tests and were showed as the means ± SD. *p < 0.05, **p < 0.01, ***p < 0.001.

**Additional file 3: Figure S4.** The changes of MCL1 regulatory molecules (Ubiquitin E3 ligases and DUBs) after adding the different concentrate LH (0, 10, 20, 40 μM). (A) The qRT-PCR verified the changes of Ubiquitin E3 ligases (β-TRCP, HUWEI, and FBXW7) and DUBs (JOSD1, DUB3, USP9X and USP13) after adding different concentrate LH (10, 20, 40 μM). DMSO was used as control. GAPDH was used as internal reference. (B) The western blotting tested the changes of Ubiquitin E3 ligases (β-TRCP, HUWEI, and FBXW7) after adding the different concentrate LH (10, 20, 40 μM). DMSO was used as control. Tubulin was used as internal reference. All data were analyzed by unpaired Student’s t-tests and were showed as the means ± SD. *p < 0.05, **p < 0.01, ***p < 0.001.

**Additional file 4: Figure S5.** Verification of BCL2-resistant-cell lines. (A) IC50 of HA14–1 in BCL2-drug-resistant cell lines (MKN-45-R, SGC-7901-R) and normal gastric cancer cell lines (MKN-45, SGC-7901). (B) The relative mRNA levels of MCL1 and BCL2 in normal gastric cancer cell lines and BCL2-drug-resistant cell lines. (C) The expression of BCL2 and MCL1 in BCL2-drug-resistant cell lines and normal gastric cancer cell lines. Tubulin was used as internal reference. All data were analyzed by unpaired Student’s t-tests and were showed as the means ± SD. *p < 0.05, **p < 0.01, ***p < 0.001.

**Additional file 5: Figure S6.** Patient information.

## Data Availability

All the data reported by the manuscript are publicly available and the materials are also freely available [51].

## Abbreviations

LH: Lycorine hydrochloride
MCL1: myeloid cell leukemia-1
BCL2: B-cell lymphoma-2
FBXW7: F-Box And WD Repeat Domain Containing 7
PDX: Patient-Derived tumor xenograft
HA14-1: (R)-ethyl 2-amino-6-bromo-4-((R)-1-cyano-2-ethoxy-2-oxoethyl)-4H- chromene-3-carboxylate
BH: B-cell lymphoma-2 homology
BCL-XL: B-cell lymphoma like protein 1
BFL-1/A1: B-cell-lymphoma-2-related protein A1
BIM: B-cell lymphoma-2 interacting mediator
BAX: B-cell lymphoma-2 Assaciated X
BID: B-cell lymphoma-2 homology-3 interacting domain death agonist
PUMA: B-cell lymphoma-2 binding component 3
NOXA: Phorbol-12-myristate-13-acetate-induced protein 1
BAD: B-cell lymphoma-2 associated agonist of cell death
BMF: B-cell lymphoma-2 modifying factor
HRK: Activator of apoptosis harakiri
BIK: B-cell lymphoma-2 interacting killer
BAK: B-cell lymphoma-2 homologous antagonist/killer
BCL2-W: B-cell lymphoma-2 like protein 2
ATCC: American Type Culture collection
BrdU: 5-bromo-2′-deoxyuridine
CDK1: Cyclin-dependent kinase 1
CDK2: Cyclin-dependent kinase 2
PARP: Poly [ADP-ribose] polymerase 1
MG132: Z-Leu-leu-leu-al
β-TrCP: F-box/WD repeat-containing protein 1A
TUNEL: Terminal Deoxynucleotidyl Transferase mediated dUTP Nick-End Labeling
RIPA: Radio Immunoprecipitation Assay
PMSF: Phenylmethanesulfonyl fluoride
CQ: Chloroquine
DAB: 3,3′-diaminobenzidine
DAPI: 4′,6-diamidino-2-phenylindole
DMEM: Dulbecco’s modified Eagle’s medium
DMSO: Dimethyl sulfoxide
LC3B: The microtubule-associated protein light chain 3 beta
IHC: Immunohistochemical
MTT: 3-(4,5-Dimethylthiazol-2-l)-2,5-Diphenyltetrazolium Bromide
NOD/SCID: Nonobese diabetic/severe combined immunodeficiency
qRT–PCR: The quantitative reverse transcription–PCR
SPF: Specific pathogen-free
