# Correction: Lycorine hydrochloride inhibits cell proliferation and induces apoptosis through promoting FBXW7-MCL1 axis in gastric cancer

**DOI:** 10.1186/s13046-022-02503-1

**Published:** 2022-10-18

**Authors:** Chongyang Li, Chaowei Deng, Guangzhao Pan, Xue Wang, Kui Zhang, Zhen Dong, Gaichao Zhao, Mengqin Tan, Xiaosong Hu, Shaomin Shi, Juan Du, Haoyan Ji, Xiaowen Wang, Liqun Yang, Hongjuan Cui

**Affiliations:** 1 State Key Laboratory of Silkworm Genome Biology, College of Biotechnology, Southwest University, #1, Tiansheng Rd., Beibei District, Chongqing, 400716 China; 2 Cancer center, Medical Research Institute, Southwest University, Chongqing, 400716 China; 3 Chongqing Engineering and Technology Research Centre for Silk Biomaterials and Regenerative Medicine, Chongqing, 400716 China; 4 Engineering Research Center for Cancer Biomedical and Translational Medicine, Southwest University, Chongqing, 400716 China; 5 Chongqing General Hospital, University of Chinese Academy of Sciences, Chongqing, 400014 China; 6 The Fifth Hospital of Shijiazhuang, Shijiazhuang, 050021 China; 7 The Third Hospital of Hebei Medical University, Shijiazhuang, 050051 China

**Correction: J Exp Clin Cancer Res 39, 230 (2020)**

**https://doi.org/10.1186/s13046-020-01743-3**

Following publication of the original article [1], the authors identified errors in Figs. 2, 6, and Figure S3, specifically:

- Figure 2C: the western blot of C-Caspase 9 in MKN-45
- Figure 2D: the western blot of C-Caspase 3
- Figure 6B: the western blot of BCL2 in SGC-7901
- Supplementary Figure 3G: the western blot of Tubulin

**Fig. 2:**
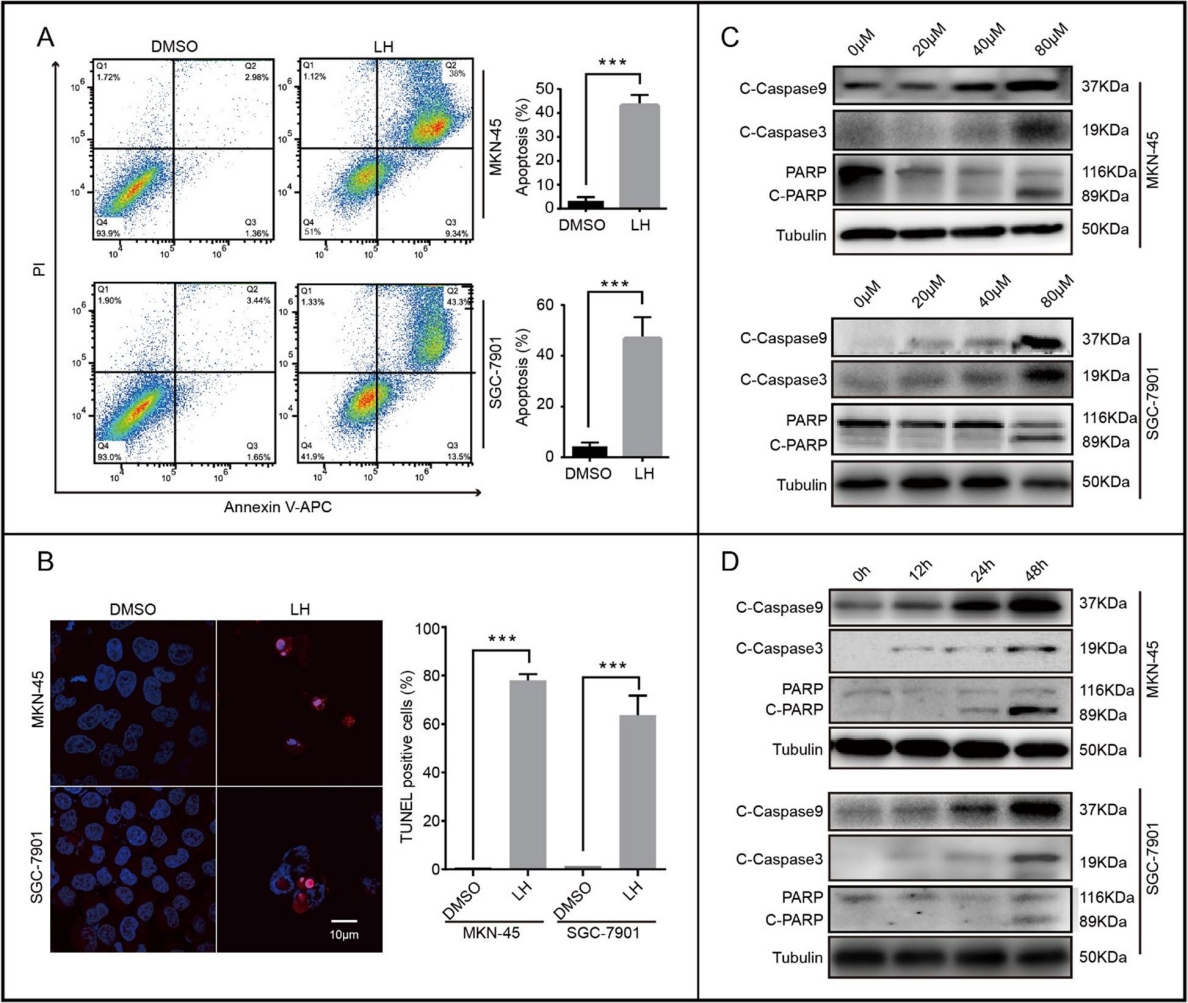
Lycorine hydrochloride induces apoptosis in gastric cancer cells. **a, b** Apoptosis of MKN-45 and SGC-7901 cells treated with 20 μM LH for 48 h were examined by flow cytometry and TUNEL staining. DMSO was used as control. **c, d** The expression of apoptotic protein, including C-Caspase 9, C-Caspase 3, PARP and cleaved PARP in gastric cancer cells treated with LH at different concentrations and time gradients. DMSO was used as control. Tubulin was used as internal reference. All data were analyzed by unpaired Student’s t-tests and were showed as the means ± SD. *p < 0.05, **p < 0.01, ***p < 0.001

**Fig. 6:**
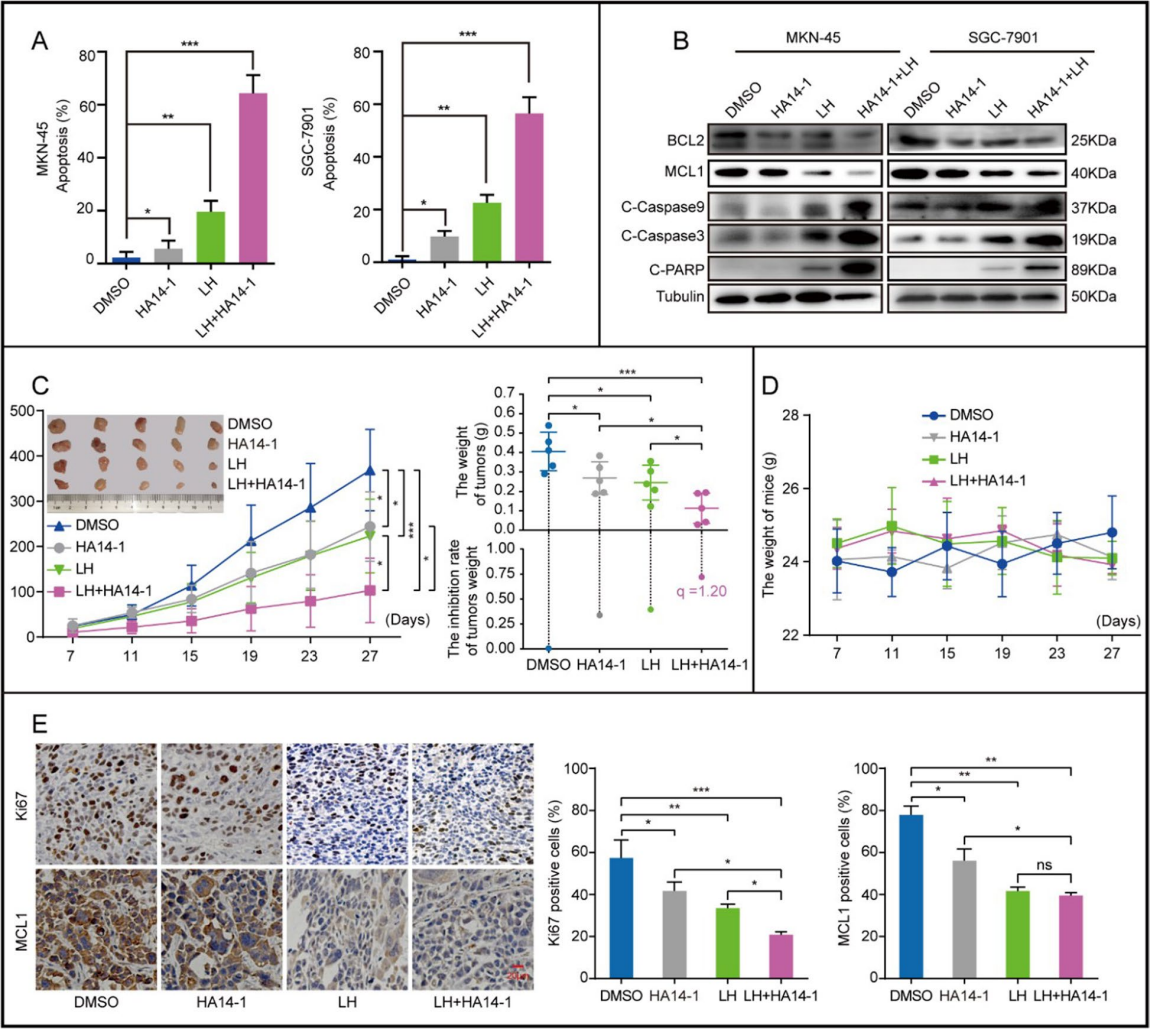
The combination of lycorine hydrochloride and HA14–1 enhances the therapeutic effect on gastric cancer. **a** Apoptosis of MKN-45 and SGC-7901 cells treated with LH (20 μM), HA14–1(9 μM) or LH (20 μM) + HA14–1(9 μM) for 48 h were examined by trypan blue staining. DMSO was used as control. Apoptotic rate of MKN-45 and SGC-7901 cells was quantified. **b** Western blotting was used to detect the expression of apoptotic protein, including BCL2, MCL1, C-Caspase 9, C-Caspase 3 and C-PARP in MKN-45 and SGC-7901 cells after 48 h of treatment with LH (20 μM), HA14–1 (9 μM) or LH (20 μM) + HA14–1 (9 μM). DMSO was used as control. **c** Tumor volume and weight of indicated mice. DMSO was used as control. The efficiency index (q) analysis of LH (30 mg/kg) combined with HA14–1 (2.5 mg/kg) treatment in the weight of PDX tumors through Jin’s formula. **d** The weight of the mice treated with DMSO, HA14–1, LH or LH + HA14–1 was measured. **e** IHC of MCL1 and Ki67 in indicated tumors. Scale bar =20 μm. Gray value of IHC positive signal in panel was quantified. All data were analyzed by unpaired Student’s t-tests and were shown as the means ± SD. *p < 0.05, **p < 0.01, ***p < 0.001

The corrected figures are given below. This correction does not change the result, interpretation, and conclusions of the study. Furthermore, the authors apologize to the readers of the journal for any inconvenience caused. The original article has been corrected.

## Supplementary Information

**Additional file 2: Figure S3G.** The expression of CDK1 and CDK2 together with MCL1 were checked in MCL1-overexpressed MKN-45 and SGC-7901 cells with 20 μM LH treatment for 48 h. DMSO and empty vector were used as control. Tubulin was used as internal reference.
