# Supplementary figures and images for: Lycorine hydrochloride inhibits cell proliferation and induces apoptosis through promoting FBXW7-MCL1 axis in gastric cancer
Source: J Exp Clin Cancer Res. 2020 Oct 30;39:230. doi: 10.1186/s13046-020-01743-3 (PMC7602321; doi:10.1186/s13046-020-01743-3)

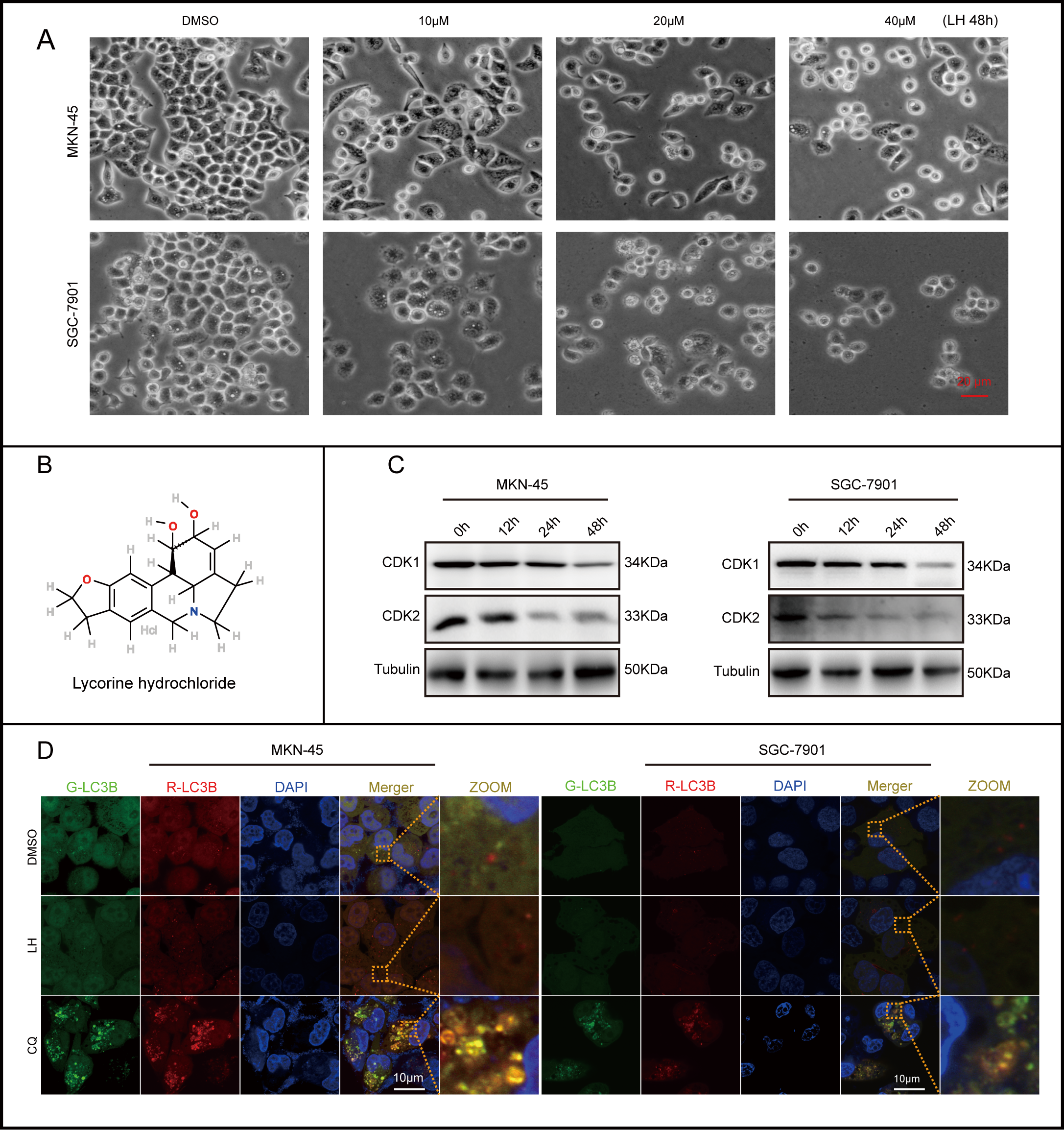

Supplement: Supplementary file 1 — Additional file 1: Figure S1. Lycorine hydrochloride inhibits cell proliferation in gastric cancer cells. (A) Morphological changes of MKN-45 and SGC-7901 cells after treatment with 10, 20, and 40 μM LH were observed. DMSO was used as control. (B) The molecular structure formula of LH. (C) The expression of CDK1 and CDK2 in gastric cancer cells after treatment with 20 μM LH for different time (0 h, 12 h, 24 h and 48 h). Tubulin was used as internal reference. (D) Autophagy flux was detected by using the mRFP-GFP-LC3-adenovirus system. CQ (Chloroquine) was used as a positive control. [file 13046_2020_1743_MOESM1_ESM.tif]

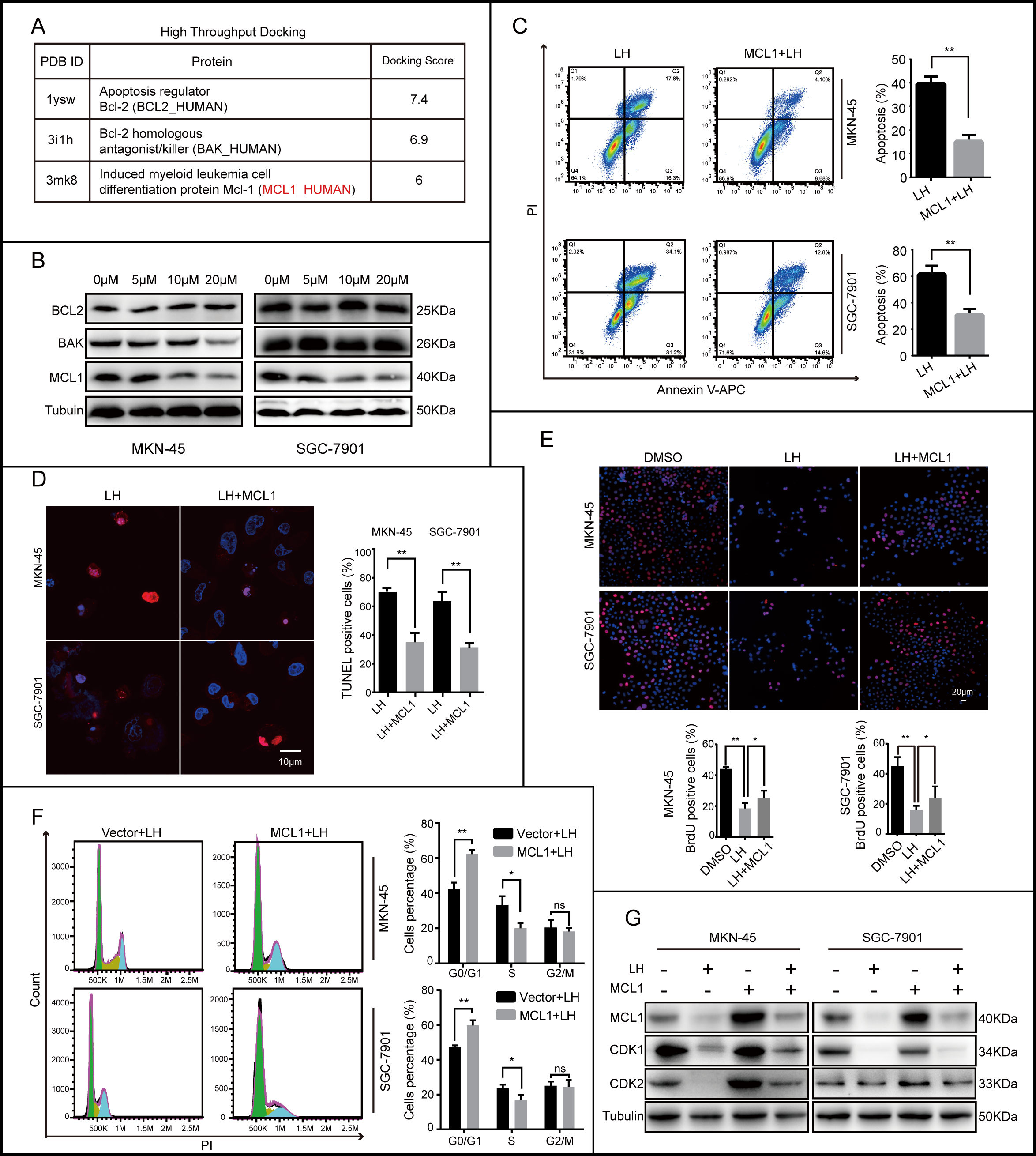

Supplement: Supplementary file 2 — Additional file 2: Figure S3. Overexpression MCL1 decreases apoptosis and restores cell proliferation induced by lycorine hydrochloride. (A) The prediction docking score of LH to its target molecules (BCL2 family) were analyzed. (B) Western blotting to verify the predicted results (LH and its target molecules). (C, D) Apoptosis was analyzed in MKN-45 and SGC-7901 cells overexpressing MCL1 after treatment with 20 μM LH for 48 h by flow cytometry and TUNEL. LH + empty vector were used as control. Apoptotic rate of MKN-45 and SGC-7901 cells in histogram was quantified. (E) BrdU-positive cells in MCL1-overexpression MKN-45 and SGC-7901 cells after treatment with 20 μM LH. DMSO and empty vector were used as control. The histograms of BrdU positive MKN-45 and SGC-7901 cells were analyzed quantitatively. (F) Cell cycle in MKN-45 and SGC-7901 cells overexpressing MCL1 after treatment with 20 μM LH for 24 h. DMSO and empty vector were used as control. Percentage of MKN-45 and SGC-7901 cells from panel at different phase was analyzed quantitatively. (G) The expression of CDK1 and CDK2 together with MCL1 were checked in MCL1-overexpressed MKN-45 and SGC-7901 cells with 20 μM LH treatment for 48 h. DMSO and empty vector were used as control. Tubulin was used as internal reference. All data were analyzed by unpaired Student’s t-tests and were showed as the means ± SD. *p < 0.05, **p < 0.01, ***p < 0.001. [file 13046_2020_1743_MOESM2_ESM.tif]

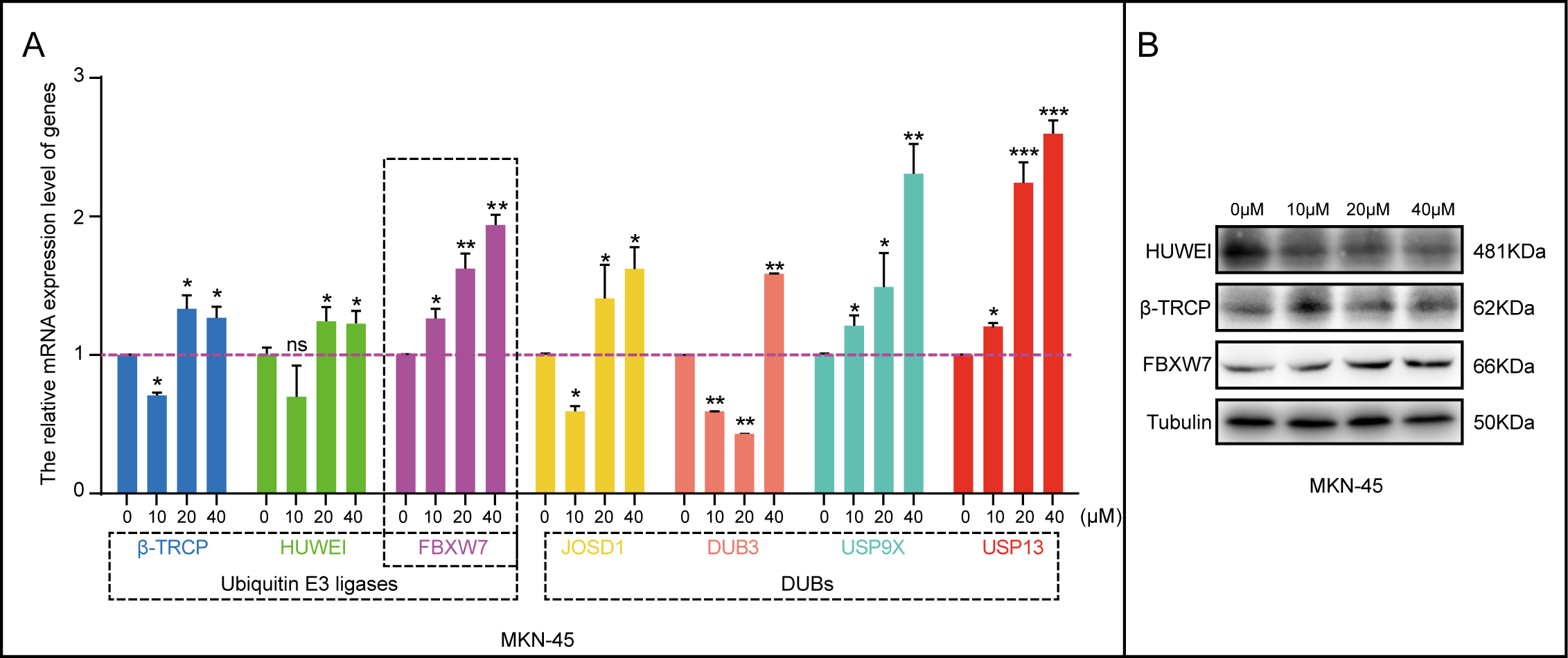

Supplement: Supplementary file 3 — Additional file 3: Figure S4. The changes of MCL1 regulatory molecules (Ubiquitin E3 ligases and DUBs) after adding the different concentrate LH (0, 10, 20, 40 μM). (A) The qRT-PCR verified the changes of Ubiquitin E3 ligases (β-TRCP, HUWEI, and FBXW7) and DUBs (JOSD1, DUB3, USP9X and USP13) after adding different concentrate LH (10, 20, 40 μM). DMSO was used as control. GAPDH was used as internal reference. (B) The western blotting tested the changes of Ubiquitin E3 ligases (β-TRCP, HUWEI, and FBXW7) after adding the different concentrate LH (10, 20, 40 μM). DMSO was used as control. Tubulin was used as internal reference. All data were analyzed by unpaired Student’s t-tests and were showed as the means ± SD. *p < 0.05, **p < 0.01, ***p < 0.001. [file 13046_2020_1743_MOESM3_ESM.tif]

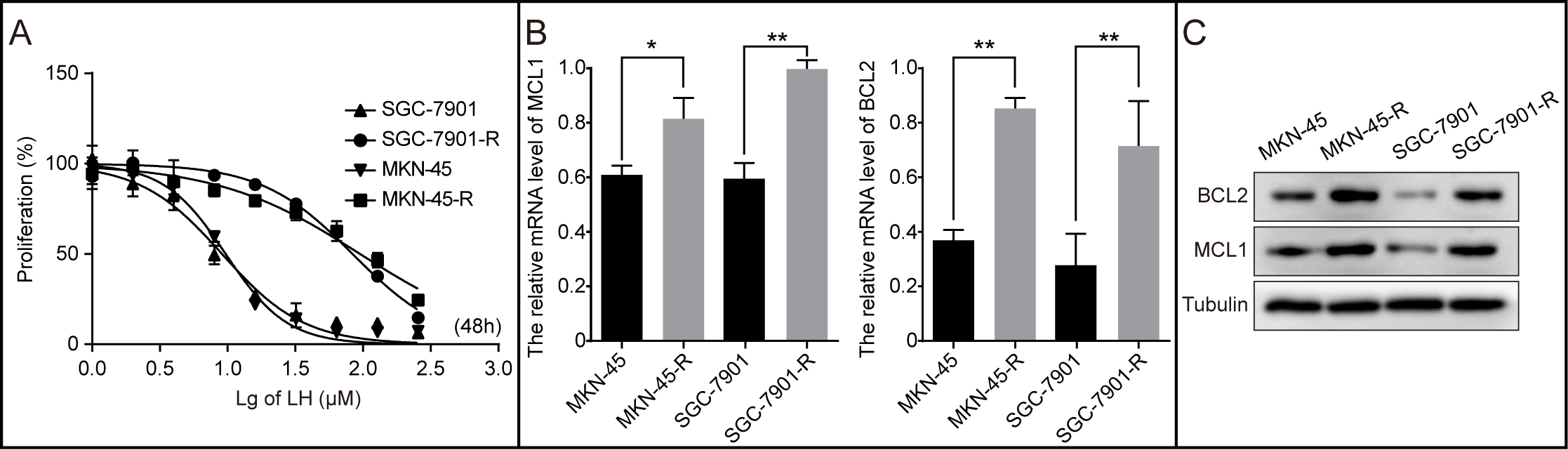

Supplement: Supplementary file 4 — Additional file 4: Figure S5. Verification of BCL2-resistant-cell lines. (A) IC50 of HA14–1 in BCL2-drug-resistant cell lines (MKN-45-R, SGC-7901-R) and normal gastric cancer cell lines (MKN-45, SGC-7901). (B) The relative mRNA levels of MCL1 and BCL2 in normal gastric cancer cell lines and BCL2-drug-resistant cell lines. (C) The expression of BCL2 and MCL1 in BCL2-drug-resistant cell lines and normal gastric cancer cell lines. Tubulin was used as internal reference. All data were analyzed by unpaired Student’s t-tests and were showed as the means ± SD. *p < 0.05, **p < 0.01, ***p < 0.001. [file 13046_2020_1743_MOESM4_ESM.tif]

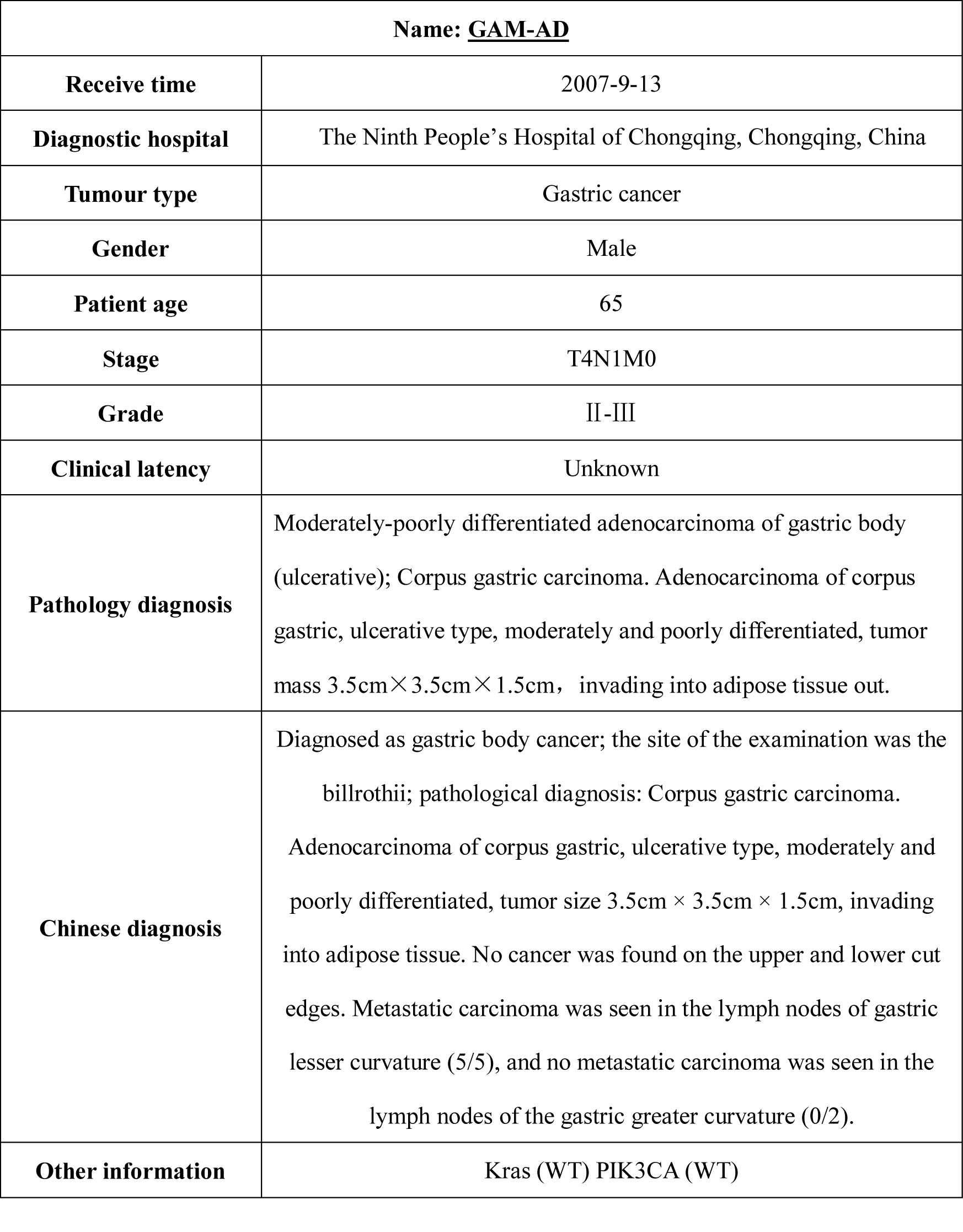

Supplement: Supplementary file 5 — Additional file 5: Figure S6. Patient information. [file 13046_2020_1743_MOESM5_ESM.tif]
