# Supplementary figures and images for: Correction: Lycorine hydrochloride inhibits cell proliferation and induces apoptosis through promoting FBXW7-MCL1 axis in gastric cancer
Source: J Exp Clin Cancer Res. 2022 Oct 18;41:306. doi: 10.1186/s13046-022-02503-1 (PMC9578188; doi:10.1186/s13046-022-02503-1)

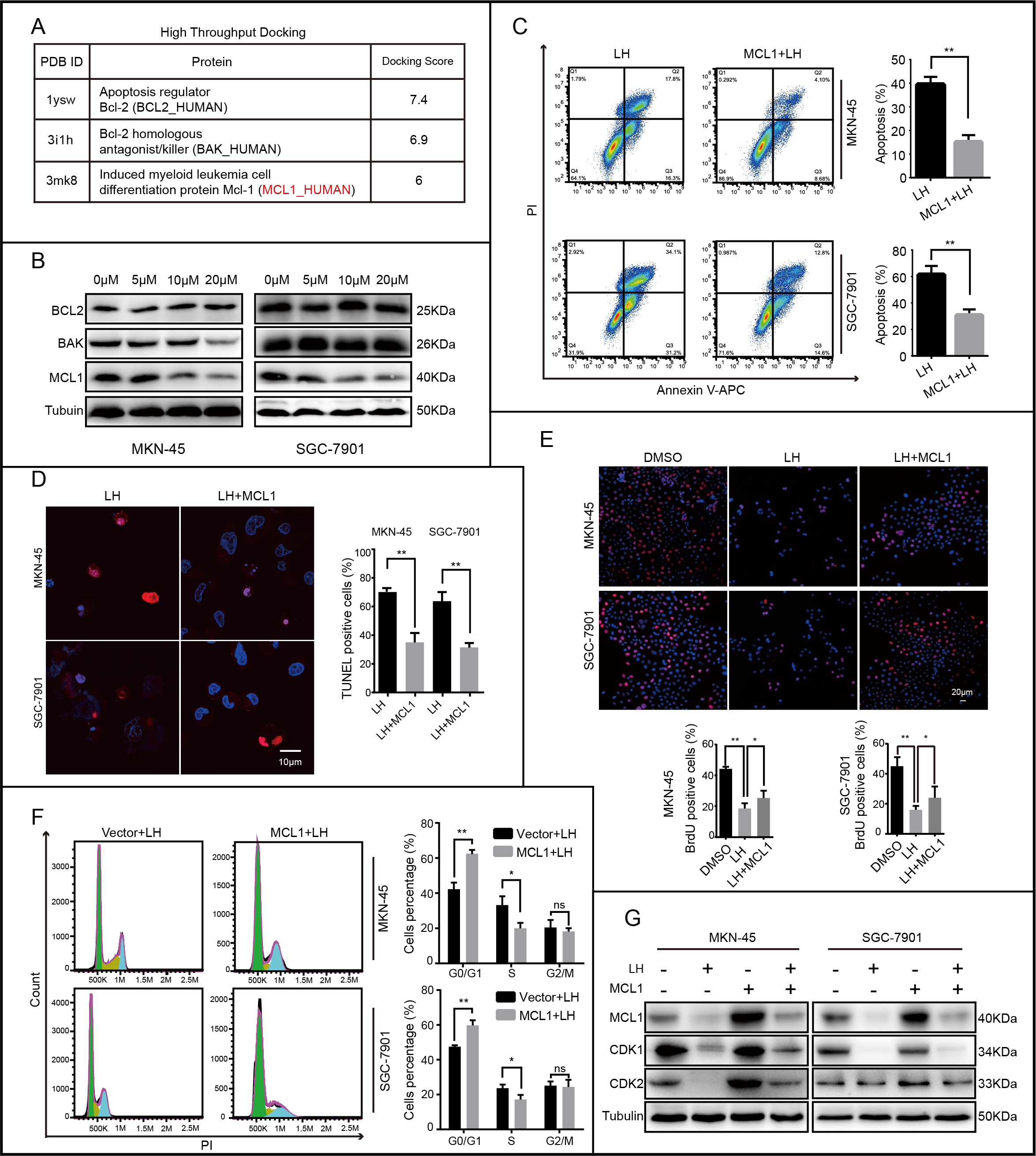

Supplement: Supplementary file 1 — Additional file 2: Figure S3G. The expression of CDK1 and CDK2 together with MCL1 were checked in MCL1-overexpressed MKN-45 and SGC-7901 cells with 20 μM LH treatment for 48 h. DMSO and empty vector were used as control. Tubulin was used as internal reference. [file 13046_2022_2503_MOESM1_ESM.tif]
